# Degradation of endogenous proteins and generation of a null-like phenotype in zebrafish using Trim-Away technology

**DOI:** 10.1186/s13059-019-1624-4

**Published:** 2019-01-23

**Authors:** Xiao Chen, Mi Liu, Hongyan Lou, Yiyi Lu, Meng-Tao Zhou, Rongying Ou, Yunsheng Xu, Kai-Fu Tang

**Affiliations:** 1 Key Laboratory of Diagnosis and Treatment of Severe Hepato-Pancreatic Diseases of Zhejiang Province, The First Affiliated Hospital of Wenzhou Medical University, Wenzhou, 325015 Zhejiang People’s Republic of China; 2 Department of Gynecology, The First Affiliated Hospital of Wenzhou Medical University, Wenzhou, 325015 Zhejiang People’s Republic of China; 3 Department of Dermato-Venereology, The First Affiliated Hospital of Wenzhou Medical University, Wenzhou, 325015 Zhejiang People’s Republic of China

**Keywords:** TRIM21, Trim-away, Protein degradation, Knockdown

## Abstract

Trim-Away is a recent technique to rapidly deplete a protein from any cell type. Guided by antibodies, TRIM21 selects proteins for destruction. However, the applicability of this method in model organisms has not been investigated. Here, we show that Trim-Away can degrade proteins in zebrafish embryos. Trim-Away depletes proteins faster than morpholinos, which enables analysis of protein function during early embryogenesis. Furthermore, Trim-Away can be applied to evaluate the role of maternally contributed proteins in zebrafish embryos. Our findings indicate that Trim-Away is a powerful tool to perform functional analysis of proteins during zebrafish development.

**Electronic supplementary material:**

The online version of this article (10.1186/s13059-019-1624-4) contains supplementary material, which is available to authorized users.

## Background

Loss of function by genomic DNA modification or mRNA targeting is a widely used strategy to investigate gene function [1–5]. TRIM21 is an E3 ubiquitin ligase that binds with high affinity to the Fc domain of antibodies and recruits the ubiquitin-proteasome system to degrade targeted proteins [6–8]. The Trim-Away technique was recently developed to directly degrade endogenous proteins in cultured cells using anti-target antibodies and TRIM21 [9]. Unlike DNA- or RNA-targeting methods, which take hours or days to deplete proteins of interest, the Trim-Away system removes endogenous proteins within minutes [9]. However, the application of Trim-Away in model organisms has not been reported.

## Results and discussion

**Fig. 1:**
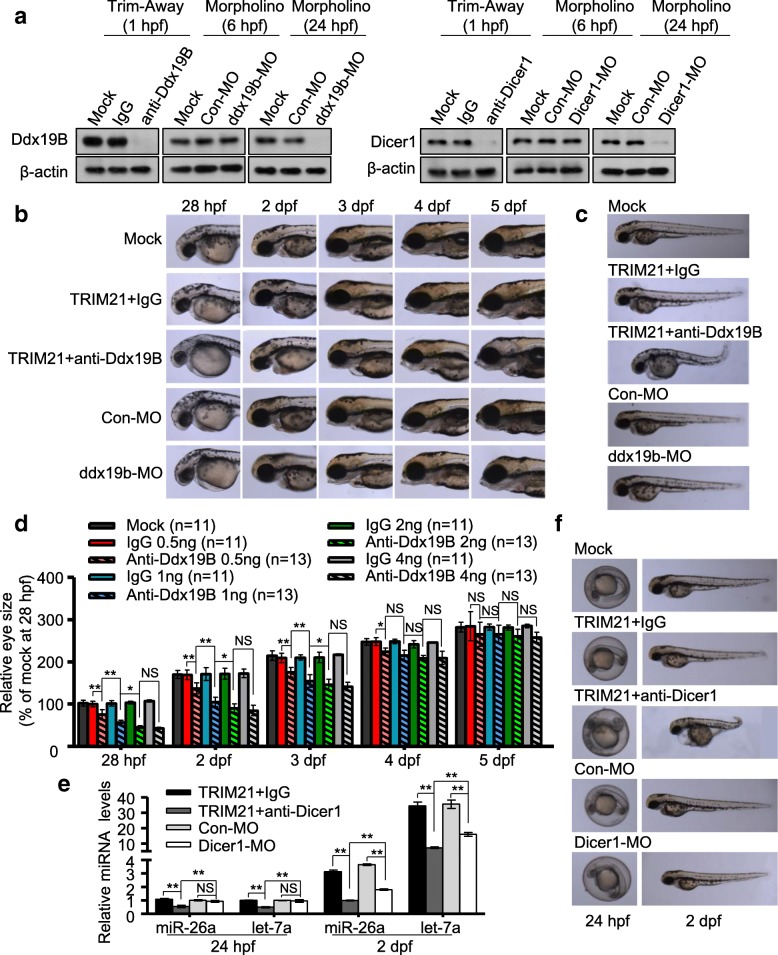
Comparing the effects of Ddx19B or Dicer1 knockdown by Trim-Away or morpholinos in zebrafish embryos. Zebrafish embryos were co-injected with TRIM21 and antibodies (anti-Ddx19B antibody, anti-Dicer1 antibody, or control IgG) or injected with morpholinos (*ddx19b*-MO, *Dicer1*-MO, or Con-MO) at 0 hpf. **a** The level of Ddx19B or Dicer1 was determined by western blotting at the indicated time points. **b** Lateral view of eye size at the indicated time points. **c** Lateral view of the body axis at 2 dpf. **d** Eye diameter in embryos injected with different doses of TRIM21/anti-Ddx19B antibody or TRIM21/IgG was measured at the indicated time points; zebrafish numbers are indicated in brackets. **e** The level of miR-26a and let-7a was determined at the indicated time points. **f** Lateral view of embryos at the indicated time points. The data in **d** and **e** are shown as the mean ± SD of three independent experiments, *P ≤ 0.05, **P ≤ 0.01, NS indicates *P* > 0.05

To determine whether the Trim-Away technique can be used to degrade proteins in zebrafish embryos in vivo, we developed transgenic zebrafish transiently expressing enhanced green fluorescent protein (EGFP) by injecting an EGFP expression plasmid into the embryo yolks at 0 h postfertilization (hpf). The human TRIM21 recombinant protein and an anti-EGFP antibody were then injected into the yolks of EGFP-expressing embryos at 24 hpf. EGFP was rapidly degraded following injection, with a half-life of 16 min (Additional file 1: Figure S1a-c). Interestingly, TRIM21 and anti-EGFP antibody were degraded concomitantly (Additional file 1: Figure S1c). EGFP expression gradually recovered when TRIM21 and anti-EGFP antibody were completely degraded (Additional file 1: Figure S1a-c). Trim-Away-induced EGFP degradation was dependent on the dose of TRIM21 and anti-EGFP antibody (Additional file 1: Figure S1d, e). Treatment with the proteasome inhibitor MG132 prevented EGFP degradation following co-injection of anti-EGFP antibody and TRIM21 (Additional file 1: Figure S1f, g), indicating proteasome-dependent degradation of target proteins in zebrafish triggered by Trim-Away. We then evaluated whether Trim-Away could be used to degrade endogenous zebrafish proteins by targeting Ddx19B, a protein essential for mRNA export, translation, and genome stability [10–12]. Injection of TRIM21 and an anti-Ddx19B antibody into the embryo yolks at 0 hpf caused Ddx19B downregulation 1 h after injection (Fig. 1a). All TRIM21/anti-Ddx19B-injected zebrafish had small eyes, and about 10% had a curved body axis (Fig. 1b, c), consistent with the findings in homozygous *ddx19b*-mutant (*ddx19b*^hi1464/hi1464^) zebrafish [13]. The reduction in eye size was most obvious during the first 2–3 days postfertilization (dpf) but gradually recovered with time and was completely restored at 5 dpf (Fig. 1b, d). The reduction in eye size was dependent on the dose of TRIM21 and anti-Ddx19B antibody (Fig. 1d). However, injection of TRIM21 and anti-Ddx19B antibody at 2 hpf or later failed to induce these morphological defects, although Ddx19B expression was still downregulated 1 h after injection (Additional file 1: Figure S2a, b). Given that Ddx19B expression was high during the first 2 h after fertilization and then gradually decreased (Additional file 1: Figure S2c, d), we proposed that Ddx19B is essential in the first 2–3 h of embryogenesis but dispensable in the late embryonic development. Morpholinos are a class of short antisense oligonucleotides widely used to knockdown gene expression in zebrafish by blocking splicing or translation of specific mRNA, thereby taking hours or days to deplete target proteins [5, 14, 15]. Microinjection of *ddx19b*-MO into the zebrafish embryo yolks at 0 hpf significantly downregulated Ddx19B expression at 24 hpf, but not at 6 hpf (Fig. 1a). Furthermore, *ddx19b*-MO failed to cause morphological defects such as small eyes and curved body axis (Fig. 1b, c). These findings indicate that morpholinos are not suitable to investigate the function of *ddx19b* because they cannot deplete Ddx19B fast enough to completely block its functional activity.

Dicer1 is a key component of the RNA interference pathway and is essential for miRNA biogenesis [16]. Although *Dicer1* mutant zebrafish retain pre-miRNA processing activity for up to 10 dpf and have no obvious defects other than the developmental delay at 7–10 dpf, morpholino-mediated Dicer1 downregulation in zebrafish causes developmental arrest at 2 dpf, indicating the importance of maternal *Dicer1* mRNA in embryogenesis [17]. Moreover, maternal-zygotic *Dicer1* mutants display earlier and more severe morphological defects than *Dicer1* morpholino-treated zebrafish [17, 18], suggesting that maternal Dicer1 protein regulates early embryogenesis. Injection of TRIM21 and an anti-Dicer1 antibody into the zebrafish yolks at 0 hpf caused faster downregulation of Dicer1 protein, earlier deregulation of pre-miRNA processing activity, and earlier and more severe morphogenesis defects compared to *Dicer1* morpholinos (Fig. 1a, e, f). Dicer1 Trim-Away zebrafish started dying at 2–3 dpf, and none were alive after 4 dpf. These data suggest that maternally contributed Dicer1 protein is essential for early embryogenesis. Our findings indicate that Trim-Away enables functional analysis of maternally contributed proteins, which are difficult to study using DNA- or RNA-targeting methods.

**Fig. 2:**
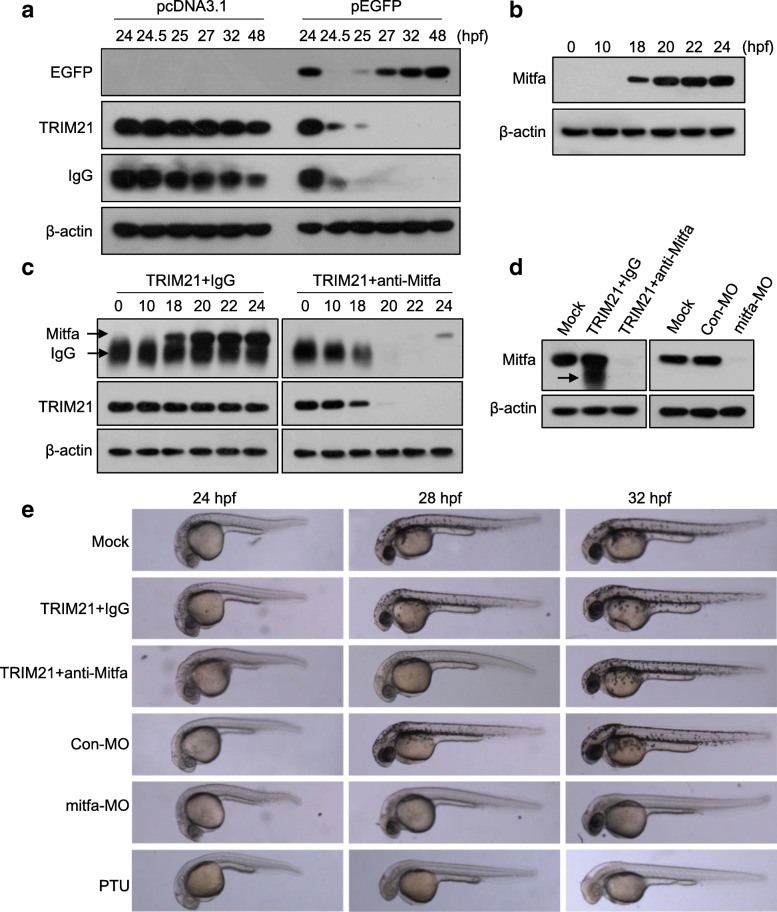
Degradation of Mitfa occurs 18 h after injection of TRIM21 and anti-Mitfa antibody. **a** EGFP expression plasmid or control pcDNA3.1 plasmid was injected into one-cell embryos. TRIM21 and anti-EGFP antibody were then co-injected into EGFP-expressing embryos or pcDNA3.1 plasmid-injected embryos at 24 hpf. The levels of EGFP, anti-EGFP antibody, and TRIM21 were analyzed by western blotting at different time points. **b** Mitfa expression in zebrafish embryos was determined at different time points by western blotting. **c** One-cell embryos were co-injected with TRIM21 and an anti-Mitfa antibody or nonspecific IgG and analyzed for the levels of Mitfa, TRIM21, and IgG heavy chain at different time points by western blotting. **d**, **e** One-cell zebrafish embryos were injected with *mitfa* morpholinos (*mitfa*-MO) or control morpholinos (Con-MO) or co-injected with TRIM21/anti-Mitfa antibody or TRIM21/nonspecific IgG. Mitfa knockdown was confirmed at 22 hpf by western blotting; the arrow indicates IgG heavy chain (**d**). Lateral view of the embryos at different time points; embryos treated with *N*-phenylthiourea (PTU) were used as positive controls (**e**)

It has been reported that TRIM21, anti-target antibodies, and target proteins are all degraded during the Trim-Away process [9]. Consistently, we found that TRIM21 and anti-EGFP antibody were rapidly degraded after co-injection into the yolks of zebrafish embryos that express EGFP (Fig. 2a). However, they degraded more slowly after co-injection into the yolks of embryos that did not express EGFP (Fig. 2a), suggesting that the injected TRIM21 and anti-target antibodies can be stored in the embryos in the absence of the target protein. Genetic manipulation in zebrafish is usually performed by microinjection at early stage embryos. Therefore, we assessed whether co-injection of TRIM21 and anti-target antibody into one-cell embryos could degrade proteins expressed several hours later. After injection of an EGFP expression plasmid into one-cell embryos, EGFP expression was detected at 6 hpf; however, co-injection with TRIM21/anti-EGFP antibody delayed the EGFP expression until 16 hpf (Additional file 1: Figure S3).

Next, we examined whether Trim-Away could be used to delay the expression of endogenous zebrafish proteins. Microphthalmia-associated transcription factor a (Mitfa) is a basic helix-loop-helix/leucine zipper transcription factor required for neural crest-derived melanocyte development [19, 20], and homozygous *mitfa* mutants lack melanophore pigmentation throughout the embryonic and larval development [19]. Consistent with the previous findings [19, 20], Mitfa expression in zebrafish started at 18 hpf (Fig. 2b). The degradation of injected TRIM21 and anti-Mitfa antibody was slow before Mitfa expression but significantly accelerated afterwards (Fig. 2c). Moreover, injection of TRIM21 and anti-Mitfa antibody into one-cell embryos delayed Mitfa expression until 24 hpf and led to slight and temporary pigmentation defects (Fig. 2d, e). Transient Mitfa knockdown using the Trim-Away technology did not produce long-term effects since the embryos were able to develop into fertile adult zebrafish without gross abnormalities. In agreement with the previous findings [21, 22], *mitfa*-MO induced more profound pigmentation defects than Trim-Away (Fig. 2e).

Methionine sulfoxide reductase B3 (Msrb3) catalyzes the reduction of methionine sulfoxide to methionine. Homozygous mutations of the *Msrb3* gene are associated with human deafness [23], and Msrb3 knockout caused profound hearing loss by inducing degeneration of stereocilia hair cells [24], whereas morpholino-induced Msrb3 knockdown compromised otolith development [25]. Therefore, we investigated whether Trim-Away could be used to analyze Msrb3 function. Msrb3 expression started at 15 hpf (Additional file 1: Figure S4a). Injection of TRIM21 and an anti-Msrb3 antibody into one-cell embryos decreased the Msrb3 expression at 18 hpf and resulted in abnormal numbers of otoliths, which were tiny and/or fused (Additional file 1: Figure S4b, c). Thus, injection of TRIM21 together with anti-target antibody into one-cell embryos can degrade proteins produced several hours later. This suggests that, in addition to degradation of disease-causing proteins, Trim-Away could be used to prevent their expression.

We next confirmed the specificity of the Trim-Away system in zebrafish. First, in a plasmid rescue assay, co-injection of a Mitfa expression plasmid with TRIM21/anti-Mitfa antibody completely restored the pigmentation phenotype (Additional file 1: Figure S5a, b). Second, injection of TRIM21 with two antibodies against different regions of Ddx19B or Dicer1 caused target protein degradation and produced similar phenotypic defects (Additional file 1: Figure S5c-f). Third, injection of TRIM21 alone or together with a control IgG did not affect the expression of EGFP, Ddx19B, Dicer1, Mitfa, and Msrb3 and failed to induce morphological abnormalities (Figs. 1 and 2c-e; Additional file 1: Figure S1a-1e, S2a-b, and S3, S4b-c, S5, S6, S8). Finally, injection of anti-target antibodies alone did not induce target protein degradation and phenotypic defects (Additional file 1: Figure S1a, S3a, and S7). However, these data do not rule out nonspecific effects, especially for the pleiotropic phenotypes of Ddx19B and Dicer1 Trim-Away embryos. To clarify pleiotropic or nonspecific effects, transcriptome analysis should be performed to compare gene expression in Trim-Away embryos with that in zygotic or maternal-zygotic mutants.

Two technical points should be considered. First, although injection of TRIM21/target antibody into either cells or yolks led to target protein degradation and caused similar phenotypic defects (Additional file 1: Figure S8), yolk injection is much easier to perform. Second, although Trim-Away induced dose-dependent degradation of EGFP and reduction of the eye size in the range of 0.5–2 ng of TRIM21 and anti-target antibodies (mixed 1:1) per embryo, dose elevation from 1 to 2 ng only slightly augmented EGFP degradation and eye size reduction and that from 2 to 4 ng did not cause further enhancement (Fig. 1d; Additional file 1: Figure S1d, e). Therefore, injection of 1 ng TRIM21 and 1 ng of antibody into the zebrafish yolks is recommended. However, given that Mitfa overexpression seems to overwhelm the effect of TRIM21/antibodies injected (Additional file 1: Figure S5a, b), more TRIM21/antibodies should be used when the targeted proteins are highly abundant.

## Conclusions

We demonstrated that Trim-Away can be used to investigate protein function during the first few hours of zebrafish embryogenesis. In contrast, morpholinos take hours or days to deplete target proteins and thus are not suitable to investigate protein function in early embryogenesis. Moreover, Trim-Away enables functional analysis of maternally contributed proteins, as well as proteins expressed several hours after injection of TRIM21/anti-target antibody into one-cell embryos. Therefore, Trim-Away is a powerful tool to determine protein functions during zebrafish development.

## Methods

Mixtures of recombinant TRIM21 and anti-target antibody were directly injected into the yolks or cells of zebrafish embryos. Gene expression levels were determined by western blotting or real-time RT-PCR. Two-tailed Student’s *t* tests were performed to evaluate the statistical significance of the results. Detailed methods are available in Additional file 1. All full-length western blots are available in Additional file 2.

## Additional files

Additional file 1: Contains supplementary methods, supplementary tables, and eight supplementary figures with legends. (PDF 1250 kb)

Additional file 2: Contains all full-length western blot images. (PDF 720 kb)

Additional file 3: Review history. (DOCX 744 kb)

## Abbreviations

dpf: Days postfertilization
EGFP: Enhanced green fluorescent protein
hpf: Hours postfertilization
Mitfa: Microphthalmia-associated transcription factor a
Msrb3: Methionine sulfoxide reductase B3
PTU: *N*-phenylthiourea
