# Supplementary material for: Degradation of endogenous proteins and generation of a null-like phenotype in zebrafish using Trim-Away technology
Source: Genome Biol. 2019 Jan 23;20:19. doi: 10.1186/s13059-019-1624-4 (PMC6343325; doi:10.1186/s13059-019-1624-4)
Supplement: Supplementary file 1 — Contains supplementary methods, supplementary tables, and eight supplementary figures with legends. (PDF 1250 kb) [file 13059_2019_1624_MOESM1_ESM.pdf]

Supplementary methods

Fish culture and manipulation

Zebrafish (*Danio rerio*, AB strain) were purchased from the China Zebrafish Resource Center (Wuhan, China). Adult fish were maintained at 28.5 °C and a 12-h/12-h light/dark cycle. If required, 0.003% N-phenylthiourea (PTU, Sigma-Aldrich, St. Louis, MO, USA) was added to the egg water at 0 hpf to prevent melanization. All zebrafish were reared and handled in accordance with the Institutional Guidelines on Animal Usage and Maintenance of Wenzhou Medical University.

The pEGFP-N3 plasmid was obtained from Clontech Laboratories, Inc. (Mountain View, CA, USA). The pMitfa plasmid, originally designated as pCS2-MT-Mitfa [1], was obtained from Addgene (Cambridge, MA, USA). The pTRIM21 plasmid was constructed by inserting the coding sequence of human *TRIM21* (NM_003141.3) into the BamHI/XhoI sites of pcDNA3.1 (Invitrogen, Carlsbad, CA, USA). The primers used to amplify human *TRIM21* cDNA were: 5′-TTAAGCTTGGTACCGAGCTCGGATCCGCCACCATGGCTTCAGCAGCACGCTTGAC-3′ and 5′-GTTTAAACGGGCCCTCTAGACTCGAGTCAATAGTCAGTGGATCCTTGTGATC-3′. Plasmids were diluted in PBS to a final concentration of 0.2 mg/mL, and 2 nL of each plasmid was directly injected into the yolks of zebrafish embryos.

Morpholinos *mitfa*-MO, 5′-CATGTTCAACTATGTGTTAGCTTC-3′; *Dicer1*-MO, (5′-CTGTAGGCCAGCCATGCTTAGAGAC-3′); *ddx19b*-MO, (5′-CCGGTCTTGATAACTCTTCTGATGA-3′); and standard control,

(5′-CCTCTTACCTCAGTTACAATTTATA-3′) were purchased from Genetools LLC (Philomath, OR, USA); *mitfa*-MO and *Dicer1*-MO have been previously described [2, 3]. Morpholinos were diluted to the concentration of 1 M in distilled H_2O, and 2 nL of each was directly injected into the yolks of zebrafish embryos.

C-MYC/DDK-tagged recombinant TRIM21 protein (OriGene, Rockville, MD, USA) was mixed with anti-target antibody or nonspecific IgG control and diluted in PBS containing 0.05% NP40; the final concentration of TRIM21 and antibody/IgG was 0.5 mg/mL each. Then, 2 nL of TRIM21/antibody or TRIM21/IgG mixture was directly injected into the yolks of zebrafish embryos unless indicated otherwise. Injection of TRIM21 or antibody alone was used as a negative control. For the rescue experiment, pMitfa or pcDNA3.1 plasmids were co-injected with TRIM21/anti-Mitfa or TRIM21/IgG. For the proteasome inhibition experiment, 25 μM MG132 (Sigma, Cat# C2211) or a corresponding amount of DMSO was injected into the yolks of zebrafish embryos.

Quantitative real-time RT-PCR

Total RNA was isolated from zebrafish embryos using TRIzol reagent (Life Technologies, Carlsbad, CA, USA). Reverse transcription was performed using the M-MLV reverse transcription kit (Promega, Madison, WI, USA) and random primers (Promega) or bulge-loop miRNA RT primers (RiboBio, Guangzhou, China). Real-time PCR was performed in an ABI 7300 instrument (Life Technologies) using the SYBR© premix Ex Taq kit (TaKaRa, Dalian, China) and the following zebrafish primers: miR-26a and let-7a (RiboBio), *actb1*, forward, 5′-CGAGCAGGAGATGGGAACC-3′ and reverse, 5′-CAACGGAAACGCTCATTGC-3′; *ddx19b*, forward, 5′-AACCTGCGGATTATGGAAGC-3′ and reverse,

5′-TCTGAAGAACCTCCACCTGG-3′.

Western blotting

Total protein was isolated from zebrafish embryos using Radio Immunoprecipitation Assay (RIPA) buffer (50 mM Tris, pH 7.4, 150 mM NaCl, 1% Triton X-100, 1% sodium deoxycholate, 2.5 mM EDTA, 0.1% SDS) supplemented with a protease inhibitor cocktail. Proteins were separated by SDS-PAGE, transferred to polyvinylidene fluoride membranes (Millipore, Danvers, MA), and incubated with primary antibodies and then with peroxidase-conjugated secondary antibodies; signals were detected with ECL Plus (GE healthcare, Piscataway, NJ, USA). To measure the levels of the injected antibodies, the membranes were directly incubated with peroxidase-conjugated secondary antibodies followed by ECL Plus detection. For most experiments, the antibodies used for western blotting were not generated in the same species as the injected antibodies, especially when the target proteins had a molecular weight similar to that of the IgG heavy chain. Thus, rabbit anti-Ddx19B antibody was used to detect the level of Ddx19B proteins in zebrafish injected with goat anti-Ddx19B antibody, whereas goat anti-Ddx19B antibody was used to detect the level of Ddx19B proteins in zebrafish injected with rabbit anti-Ddx19B antibody. However, only one anti-Mitfa antibody raised in rabbits was available; therefore, we had to use the same antibody to inject zebrafish and detect the Mitfa protein. Consequently, goat anti-rabbit IgG used as secondary antibody could detect not only the anti-Mitfa antibody bound to Mitfa proteins, but also the injected anti-Mitfa antibody. Since the molecular weight of Mitfa protein is similar to that of IgG heavy chain, the band corresponding to the Mitfa protein is very close to that corresponding to the IgG heavy chain (Fig. 2c, d; Additional file 1: Figures

S5b and S7a). Antibodies used in this study are shown in supplementary table S1.

Statistical analysis

All experimental data are presented as the mean ± standard deviation (SD) of at least three independent experiments. Two-tailed Student's *t*-tests were performed using MS Excel, and *p*-values ≤ 0.05 were considered statistically significant.

References

1. Lister JA, Robertson CP, Lepage T, Johnson SL, Raible DW. nacre encodes a zebrafish microphthalmia-related protein that regulates neural-crest-derived pigment cell fate. Development. 1999;126:3757-3767.
2. Wienholds E, Koudijs MJ, van Eeden FJ, Cuppen E, Plasterk RH. The microRNA-producing enzyme Dicer1 is essential for zebrafish development. Nat Genet. 2003;35:217-218.
3. Dooley CM, Mongera A, Walderich B, Nusslein-Volhard C. On the embryonic origin of adult melanophores: the role of ErbB and Kit signalling in establishing melanophore stem cells in zebrafish. Development. 2013;140:1003-1013.

Supplementary table S1. The Antibodies used in this study

Antibodies	SOURCE	IDENTIFIER
Rabbit anti-Dicer1 antibody	CST	catalog #3363
Mouse anti-Dicer1 antibody	Abcam	catalog #ab14601
Rabbit anti-Mitfa antibody	Genetex	catalog #GTX128263
Rabbit anti-Msrb3 Antibody	ProteinTech	catalog #14251-1-AP
Goat anti-Ddx19B antibody	Abcam	catalog #ab63946
Rabbit anti-Ddx19B antibody	OriGene	catalog #TA345740
Rabbit anti-EGFP antibody	ProteinTech	catalog #50430-2-AP
Mouse anti-EGFP antibody	ProteinTech	catalog #66002-1-Ig
Rabbit anti-TRIM21 antibody	CST	catalog #92043
Mouse anti-DDK antibody*	OriGene	catalog #TA50011
Mouse anti-beta Actin antibody	ProteinTech	catalog #66009-1-Ig
Rabbit anti-beta Actin antibody	ProteinTech	catalog #20536-1-AP
Normal goat IgG control	R&D	catalog #AB-108-C
Normal rabbit IgG control	Millipore	catalog #12-370
Normal mouse IgG control	Millipore	catalog #12-371
Goat anti-rabbit IgG(H+L)secondary antibody, HRP conjugate	Boster	catalog #BA1054
Goat anti-mouse IgG(H+L)secondary antibody, HRP conjugate	Boster	catalog #BA1050
Rabbit anti-goat IgG(H+L)secondary antibody, HRP conjugate	Boster	catalog #BA1060

* anti-DDK antibody was used to detect the C-MYC/DDK tagged TRIM21 recombinant protein

Supplementary figures

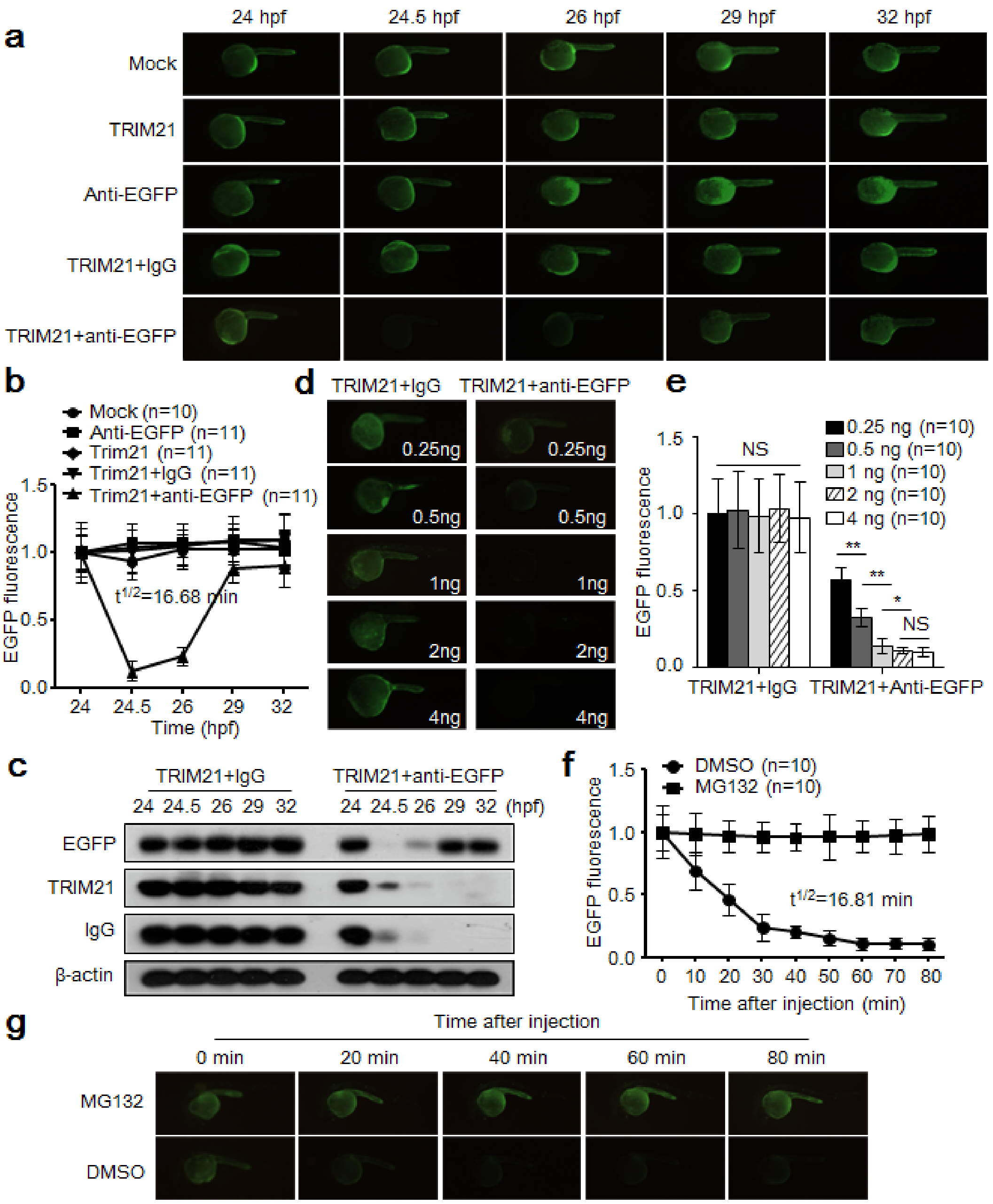

Figure S1. Acute degradation of EGFP by Trim-Away in zebrafish embryos.

The pEGFP-N3 plasmid was injected into one-cell zebrafish embryos to establish EGFP-expressing transgenic zebrafish. **a-c** EGFP-expressing zebrafish embryos were co-injected with TRIM21 and an anti-EGFP antibody or nonspecific IgG at 24 hpf and analyzed at different time points for the levels of EGFP, TRIM21, and IgG heavy chain. Mock injection and injection of TRIM21 or anti-EGFP antibody alone were used as negative

controls. Representative microscopy images (**a**), mean fluorescence intensity (**b**), and representative western blotting images of EGFP protein, TRIM21, and IgG heavy chain (**c**) are shown. **d, e** EGFP-expressing zebrafish embryos were co-injected with different doses of TRIM21 and an anti-EGFP antibody or nonspecific IgG at 24 hpf and analyzed for EGFP expression at 24.5 hpf. Representative microscopy images (**d**) and mean fluorescence intensity (**e**) are shown. **f, g** EGFP-expressing zebrafish embryos were co-injected with TRIM21/anti-EGFP antibody together with MG132 or DMSO at 24 hpf and analyzed for EGFP expression at different time points; mean fluorescence intensity (**f**) and representative microscopy images (**g**) are shown. The data in (**b**, **e**, **f**) are presented as the mean ±SD of three independent experiments; zebrafish numbers are indicated in brackets. $^{*}P \leq 0.05$ and $^{**}P \leq 0.01$; NS, not significant ($P > 0.05$).

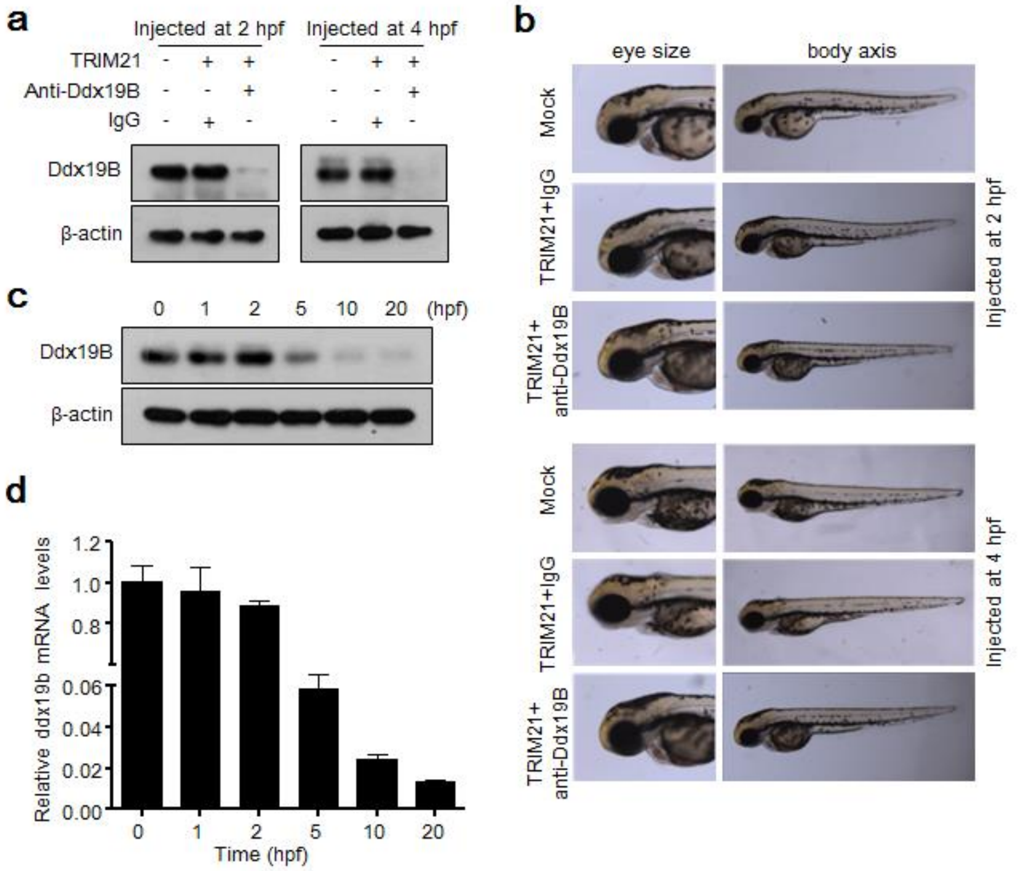

Figure S2. Injection of TRIM21 and anti-Ddx19B antibody at 2 hpf or later failed to induce morphological defects.

a, b Zebrafish embryos were co-injected with TRIM21 and an anti-Ddx19B antibody or IgG control at the indicated time points. Ddx19B level was determined by western blotting 1 hour after injection (**a**). Lateral view of the eye and body axis of embryos at 2 dpf (**b**). **c, d** Ddx19B expression in zebrafish embryos was determined at different time points by western blotting (**c**) and real-time RT-PCR (**d**).

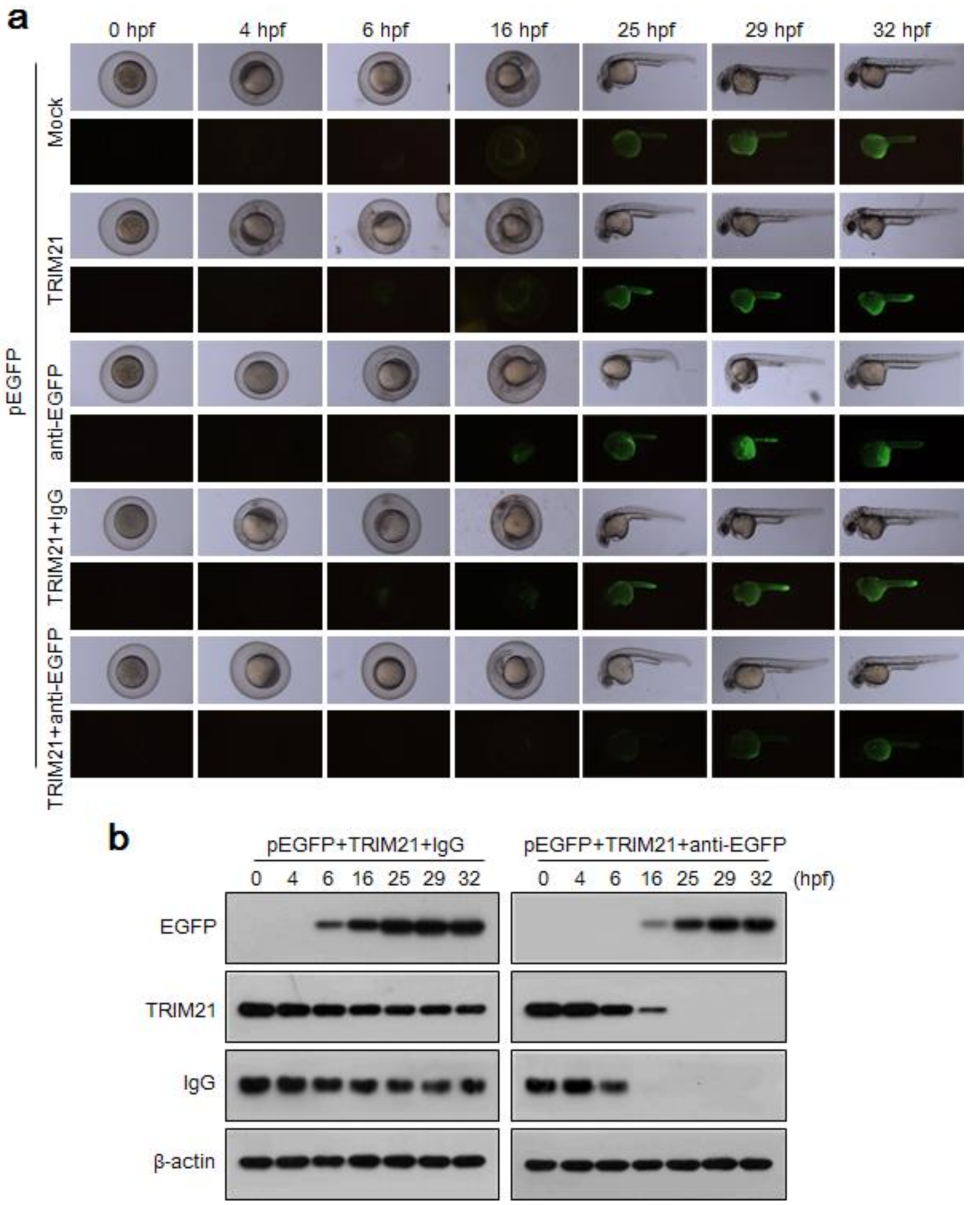

Figure S3. EGFP degradation occurs 6 hours after the injection of TRIM21 and anti-EGFP antibody.

One-cell embryos were co-injected with the EGFP expression plasmid, TRIM21, and anti-EGFP antibody or nonspecific IgG and analyzed for the levels of EGFP, TRIM21, and IgG heavy chain at different time points. Representative microscopy images (**a**) and western blotting results (**b**) are shown. Embryos injected with pEGFP alone, pEGFP/TRIM21, or pEGFP/anti-EGFP antibody were used as negative controls.

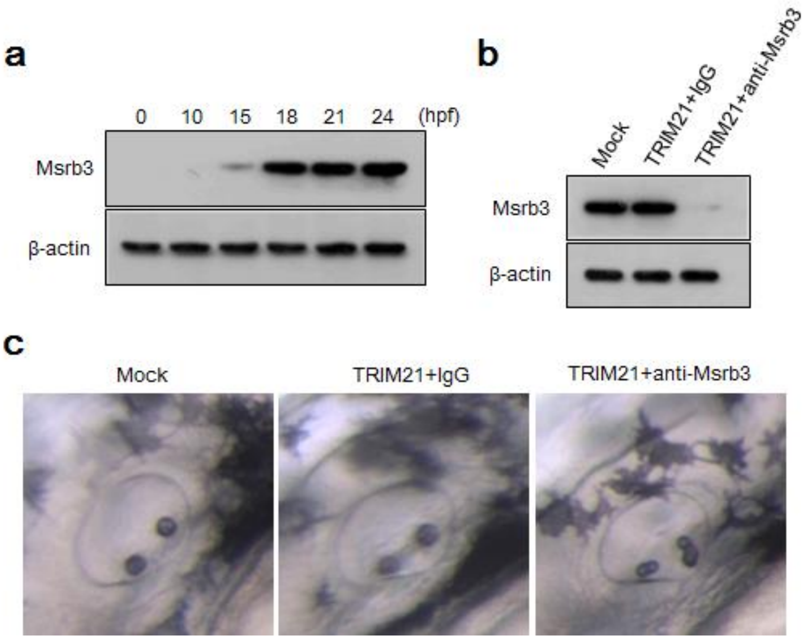

Figure S4. Injection of TRIM21 and anti-Msrb3 antibody into zebrafish embryos caused otolith defects.

a Msrb3 expression in zebrafish embryos was determined at different time points by western blotting. **b, c** One-cell embryos were co-injected with TRIM21 and anti-Msrb3 antibody or nonspecific IgG and analyzed for Msrb3 levels at 18 hpf by western blotting (**b**) or for otolith morphology at 28 hpf by stereomicroscopy (**c**).

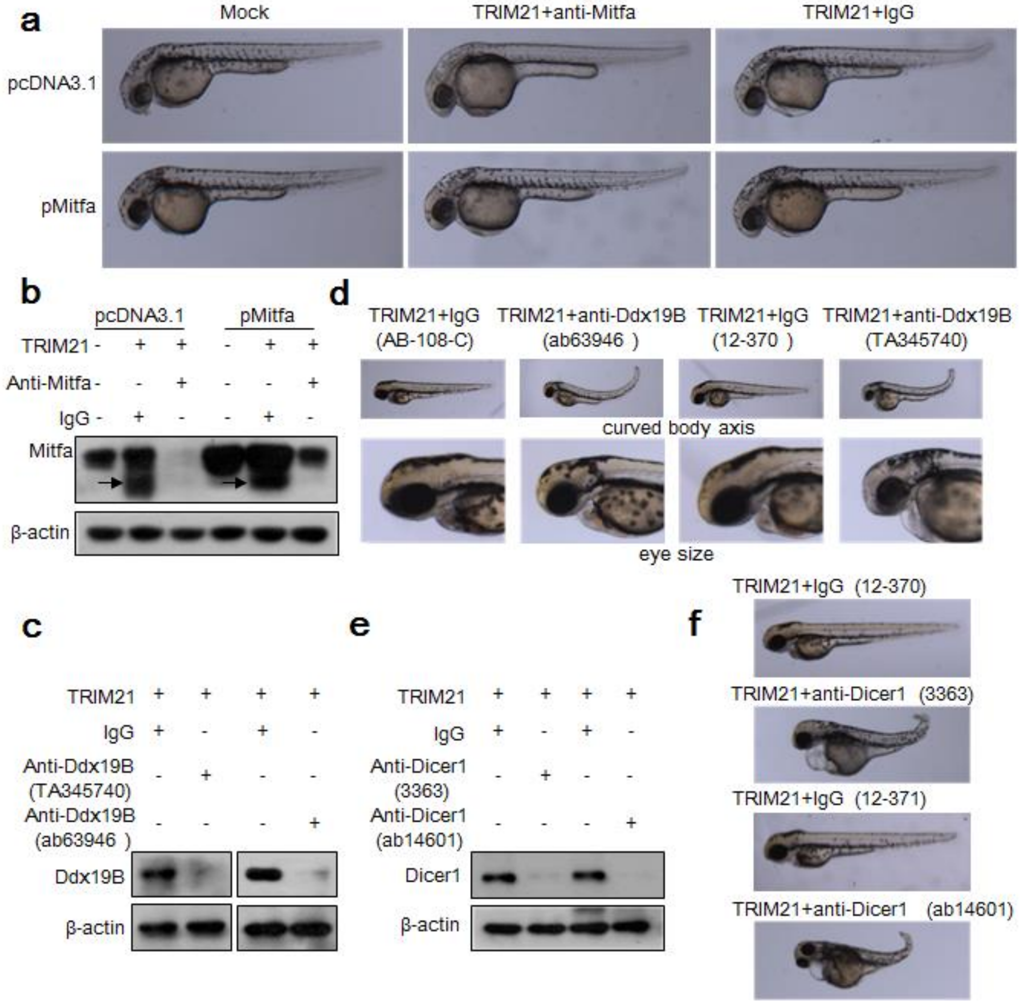

Figure S5. Specificity of Trim-Away assays in zebrafish

a, b pMitfa or pcDNA3.1, TRIM21, and anti-Mitfa antibody or control IgG were injected into the yolks of zebrafish embryos at 0 hpf as indicated. Lateral view of different embryos at 28 hpf (**a**). Mitfa protein levels were determined at 22 hpf, by western blotting; the arrow indicates the IgG heavy chain (**b**). **c, d** Zebrafish embryos were co-injected with TRIM21 and two different anti-Ddx19B antibodies or the corresponding control IgG at 0 hpf. Ddx19B protein levels were determined at 1 hpf (**c**). Lateral view of the eyes and body axis at 2 dpf (**d**). **e, f** Zebrafish embryos were co-injected with TRIM21 and two different anti-Dicer1 antibodies or the corresponding control IgG at 0 hpf. Dicer1 protein levels were determined at 1 hpf (**e**). Lateral view of the embryos at 2 dpf (**f**).

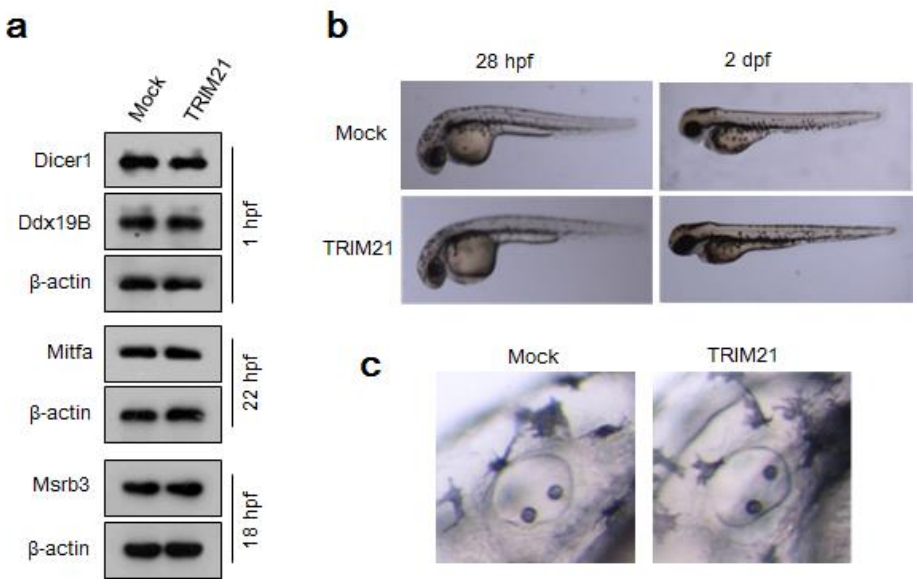

Figure S6. Injection of TRIM21 into zebrafish embryos did not affect the expression of Dicer1, Ddx19B, Mitfa, and Msrb3, and did not cause phenotypic defects.

One-cell embryos were injected with TRIM21 or mock-injected. **a** The levels of Dicer1, Ddx19B, Mitfa, and Msrb3 were determined by western blotting at the indicated time points. **b** Lateral views of embryos at 28 hpf and 2 dpf. **c** Morphology of otoliths analyzed at 28 hpf by stereomicroscopy.

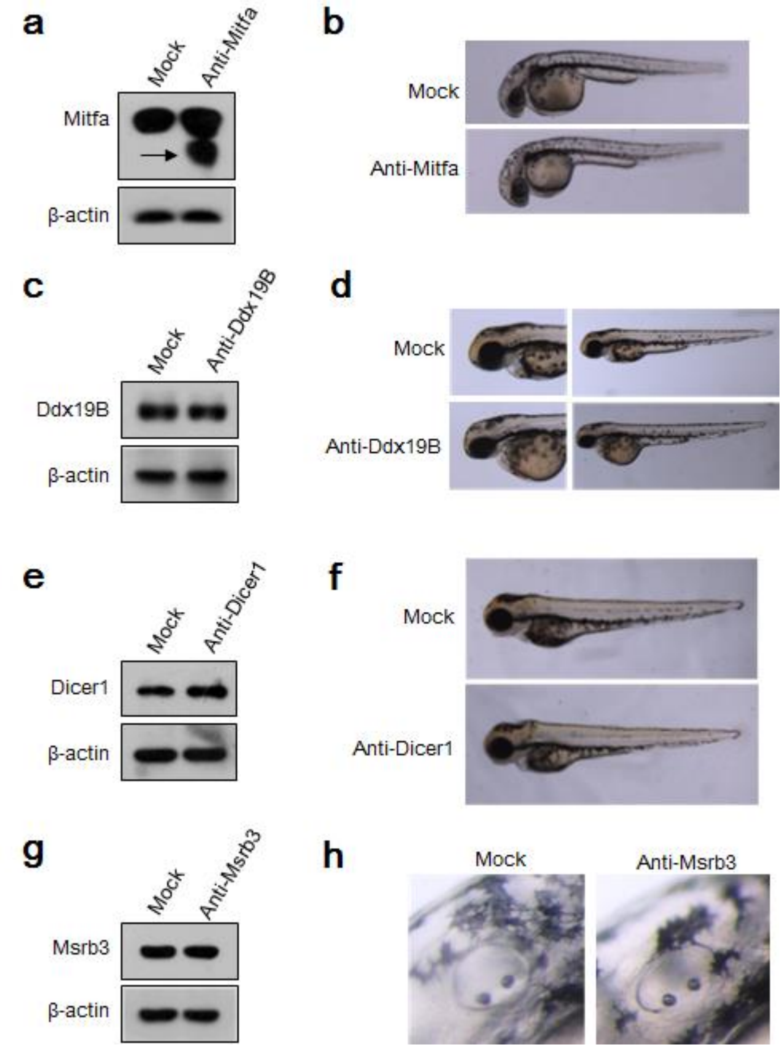

Figure S7. Injection of antibodies into one-cell embryos did not affect target protein expression and did not cause phenotypic defects.

a, b One-cell zebrafish embryos were injected with anti-Mitfa antibody or mock-injected and analyzed for the level of Mitfa at 22 hpf by western blotting; the arrow indicates the IgG heavy chain (**a**). Lateral view of embryos at 28 hpf (**b**). **c, d** One-cell zebrafish embryos were injected with anti-Ddx19B antibody or mock-injected and analyzed for the level of Ddx19B at 1 hpf by western blotting (**c**). The lateral view of embryos at 2 dpf (**d**). **e, f** One-cell zebrafish embryos were injected with anti-Dicer1 antibody or mock-injected and analyzed for the level of Dicer1 at 1 hpf by western blotting (**e**). Lateral view of embryos at 2 dpf (**f**). **g, h** One-cell

zebrafish embryos were injected with anti-Msrb3 antibody or mock-injected and analyzed for the level of Msrb3 at 18 hpf by western blotting (**g**) and for morphology of otoliths at 28 hpf by stereomicroscopy (**h**).

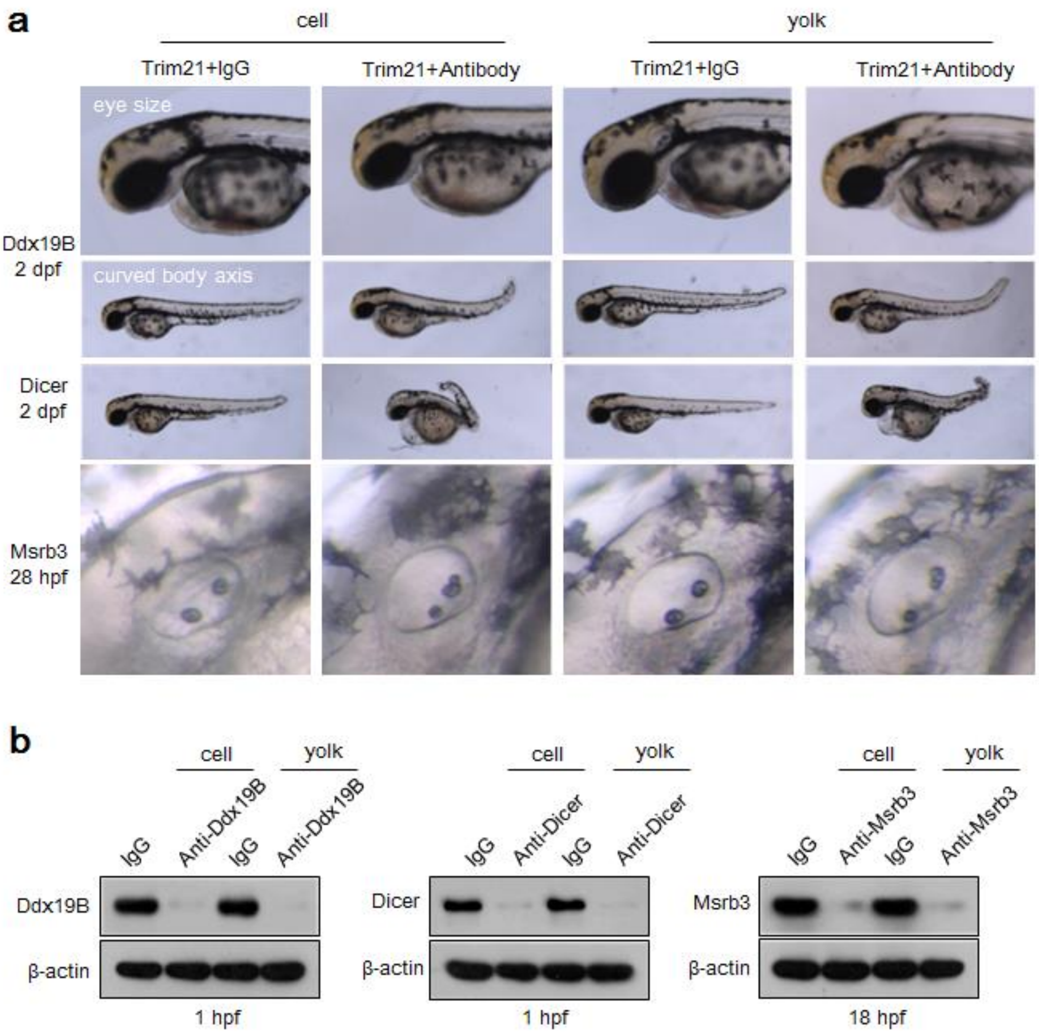

Figure S8. Comparison of the effects of yolk injection and cell injection.

TRIM21 and different antibodies were injected into cells or yolks of zebrafish at one-cell stage, and phenotypic defects and expression levels of the target proteins were examined at the indicated time points.
