# Supplementary material for: Degradation of endogenous proteins and generation of a null-like phenotype in zebrafish using Trim-Away technology
Source: Genome Biol. 2019 Jan 23;20:19. doi: 10.1186/s13059-019-1624-4 (PMC6343325; doi:10.1186/s13059-019-1624-4)
Supplement: Supplementary file 2 — Contains all full-length western blot images. (PDF 720 kb) [file 13059_2019_1624_MOESM2_ESM.pdf]

Full-length western blot images

Fig. 1

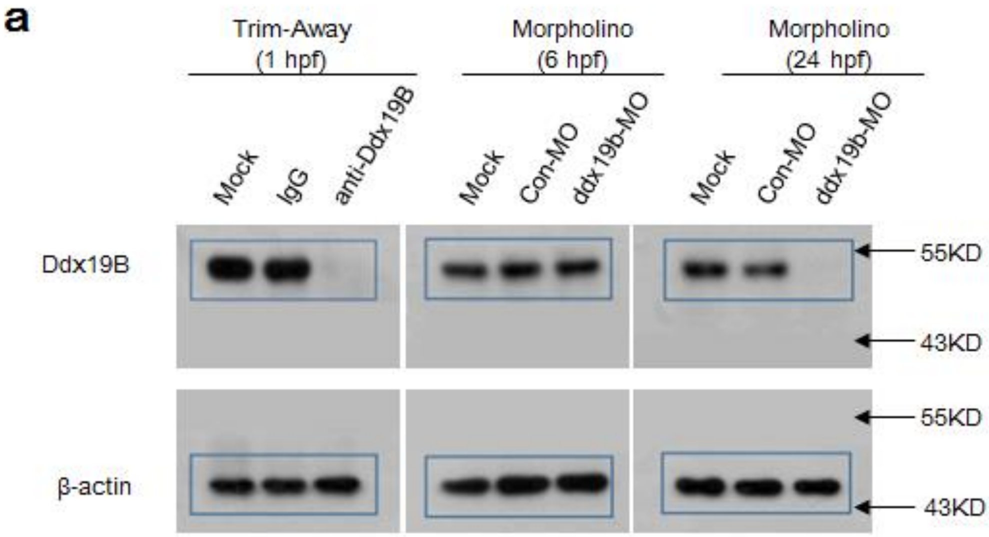

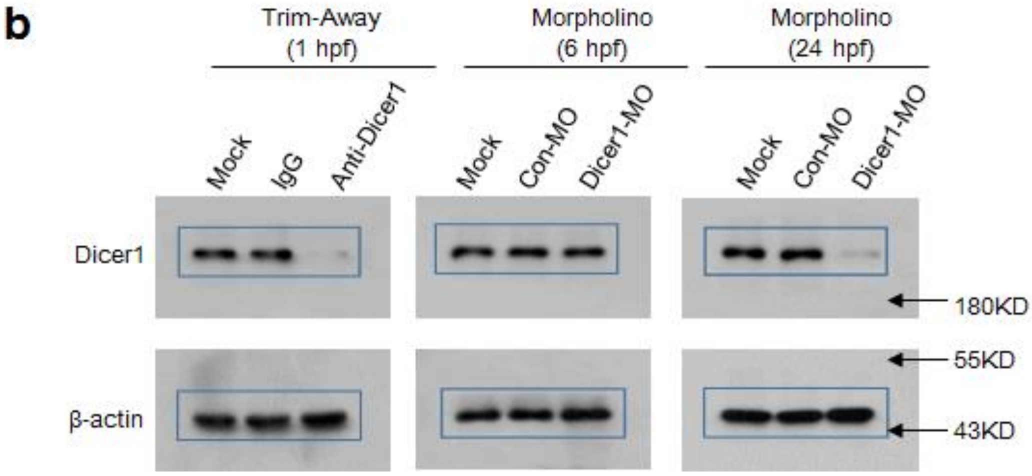

Fig. 2

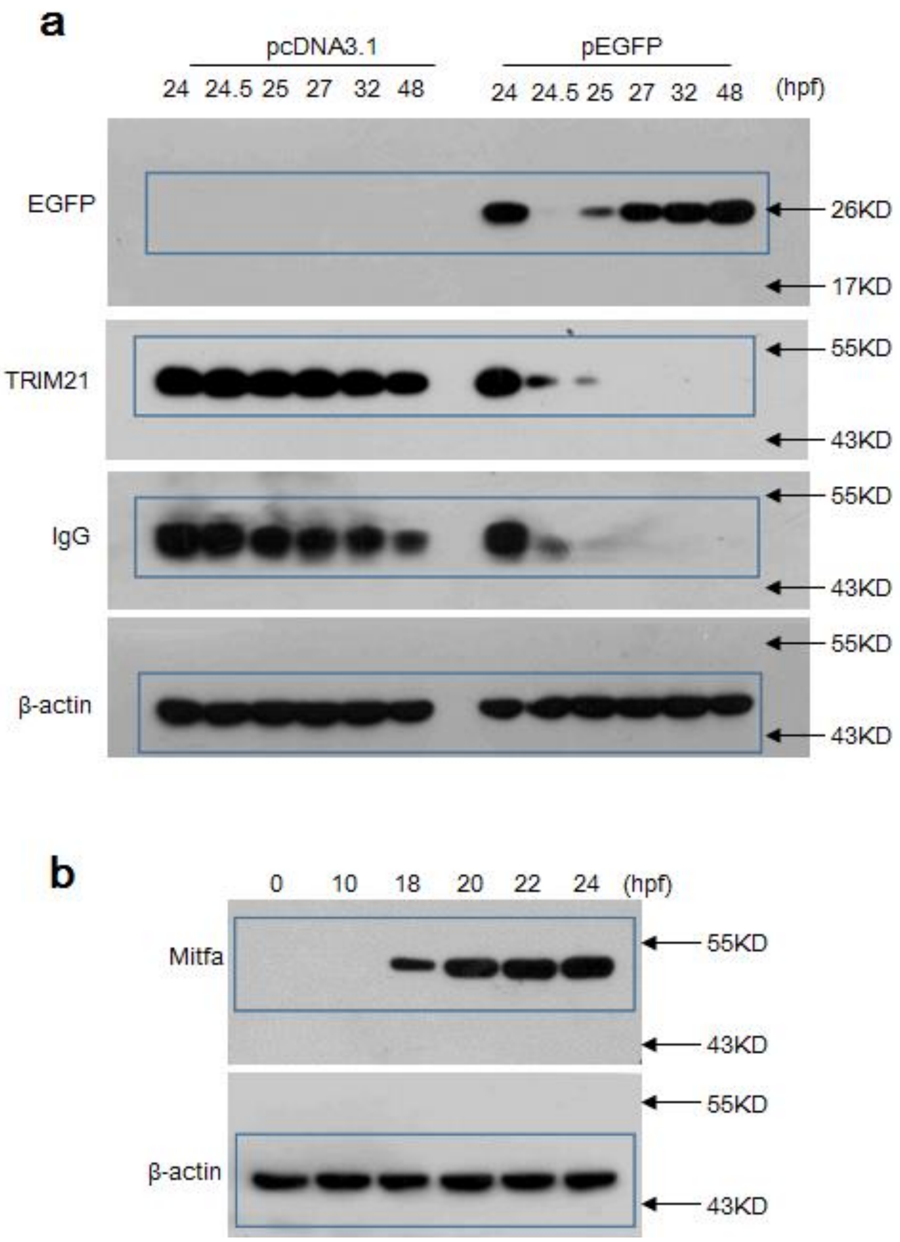
a
pcDNA3.1
pEGFP
24 24.5 25 27 32 48
24 24.5 25 27 32 48 (hpf)
EGFP
26KD
17KD
TRIM21
55KD
43KD
IgG
55KD
43KD
β-actin
55KD
43KD
b
0 10 18 20 22 24 (hpf)
Mitfa
55KD
43KD
β-actin
55KD
43KD

Fig. 2

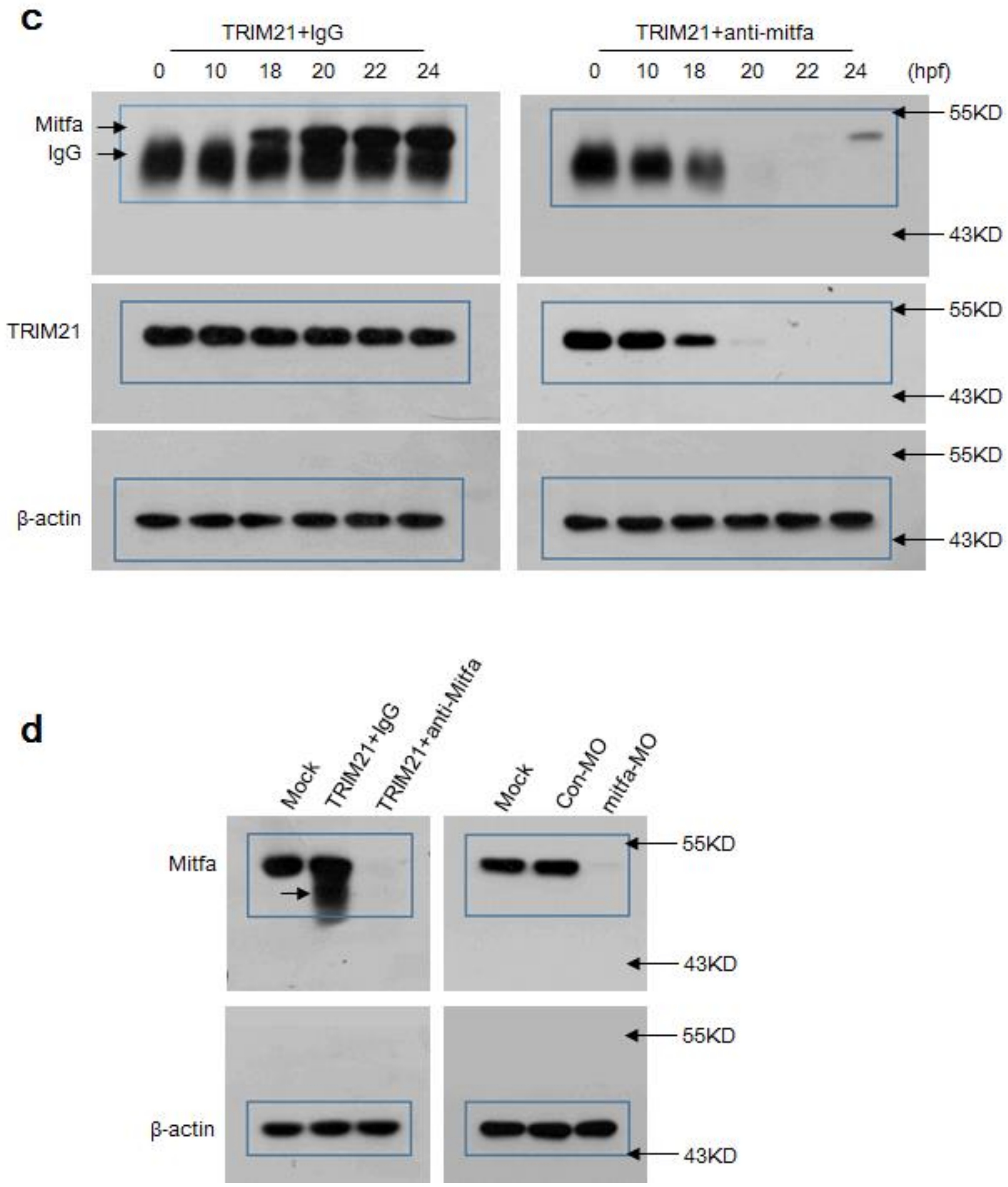
c
TRIM21+IgG
0 10 18 20 22 24
TRIM21+anti-mitfa
0 10 18 20 22 24 (hpf)
Mitfa
IgG
55KD
43KD
TRIM21
55KD
43KD
β-actin
55KD
43KD
d
Mock
TRIM21+IgG
TRIM21+anti-Mitfa
Mock
Con-MO
mitfa-MO
Mitfa
55KD
43KD
β-actin
55KD
43KD

Figure S1

c

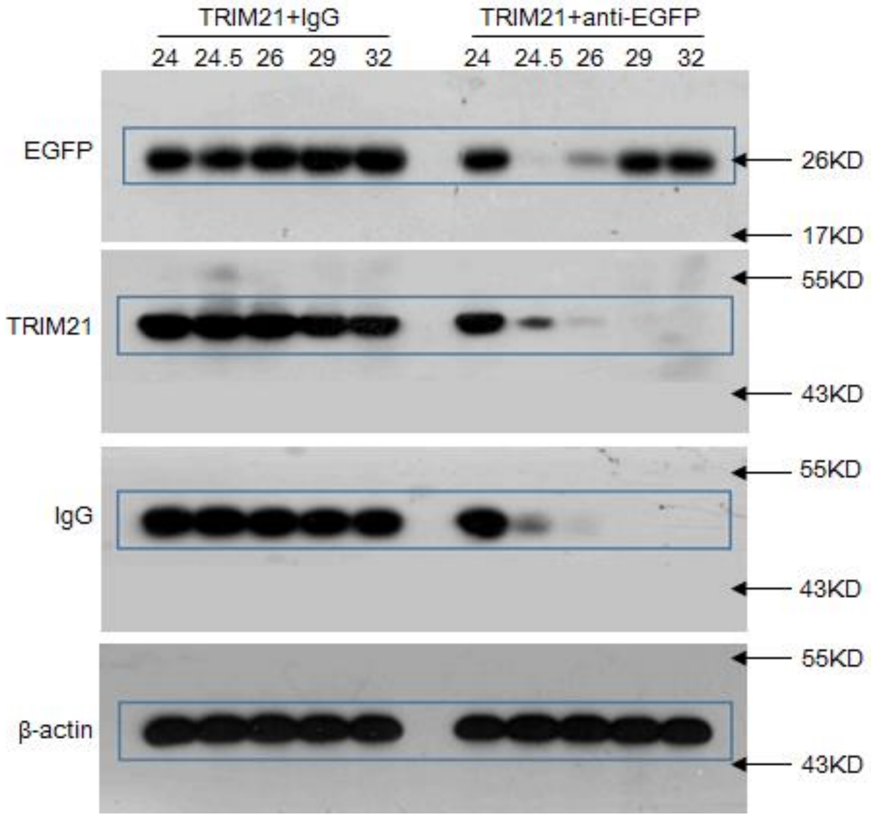
TRIM21+IgG
TRIM21+anti-EGFP
24 24.5 26 29 32
24 24.5 26 29 32
EGFP
TRIM21
IgG
β-actin
26KD
17KD
55KD
43KD
55KD
43KD
55KD
43KD

Figure S2

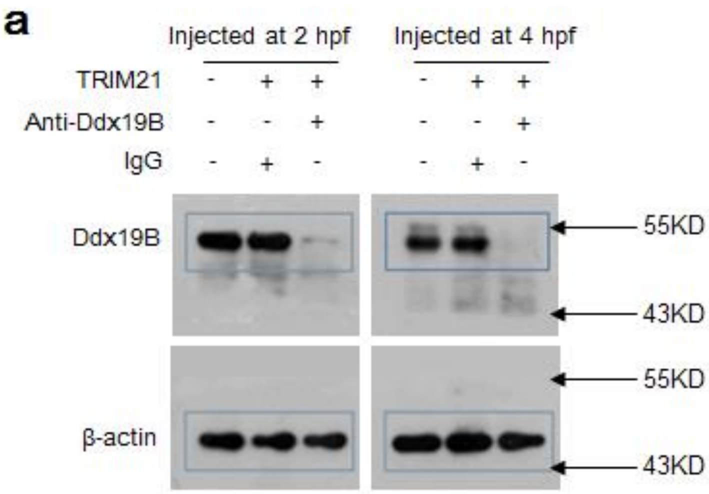
a
Injected at 2 hpf
Injected at 4 hpf
TRIM21 - + + - + +
Anti-Ddx19B - - + - - +
IgG - + - - + -
Ddx19B
β-actin
55KD
43KD
55KD
43KD

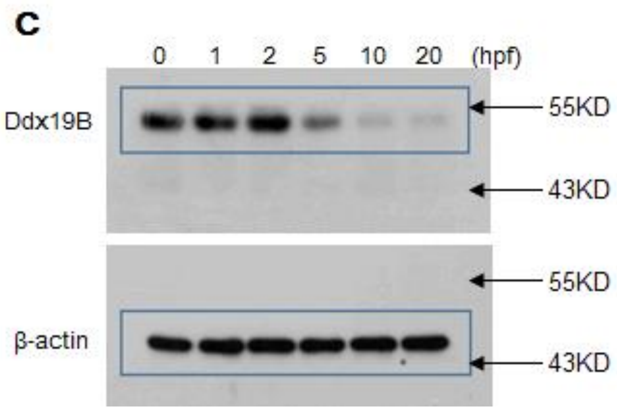
c
0 1 2 5 10 20 (hpf)
Ddx19B
β-actin
55KD
43KD
55KD
43KD

Figure S3

b

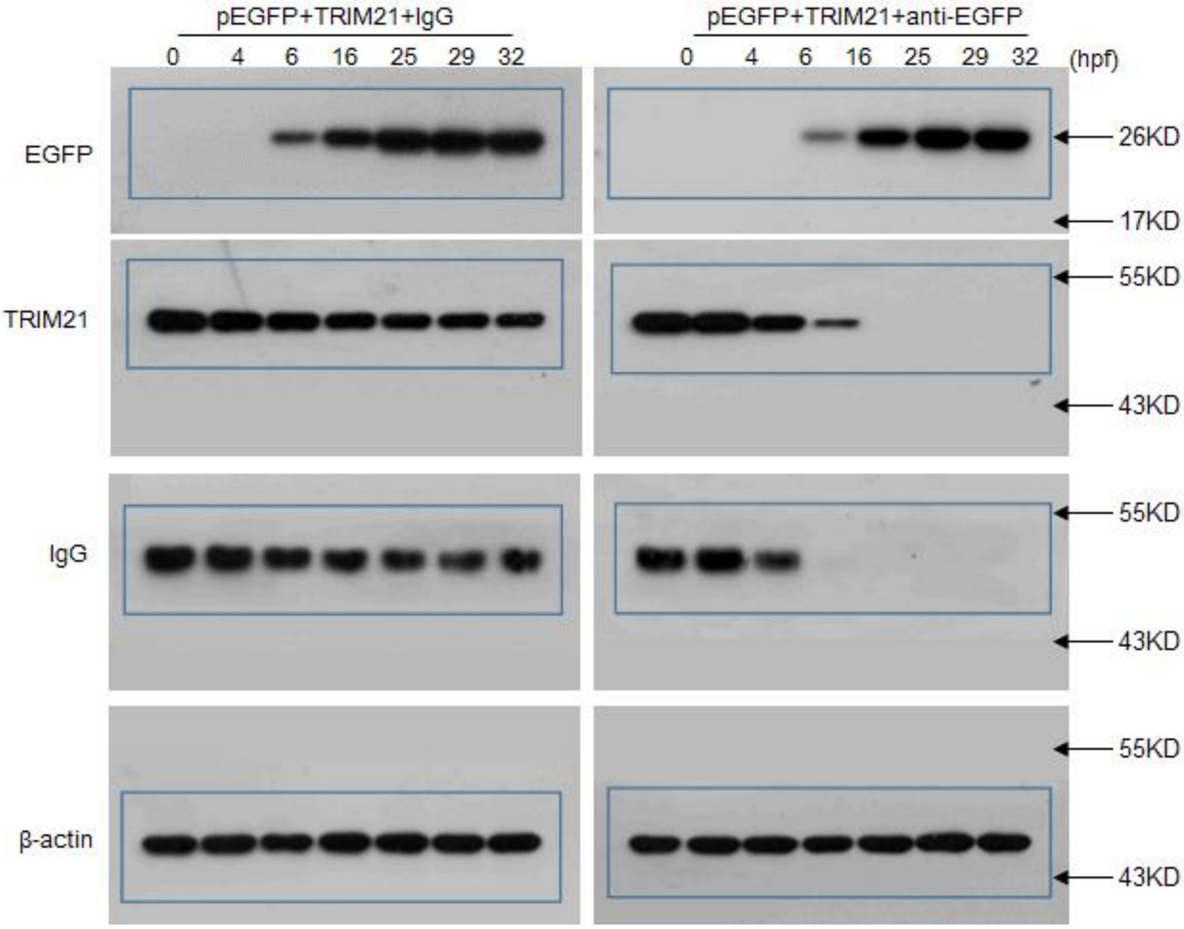

pEGFP+TRIM21+IgG
pEGFP+TRIM21+anti-EGFP
0 4 6 16 25 29 32
0 4 6 16 25 29 32
(hpf)
EGFP
TRIM21
IgG
β-actin
26KD
17KD
55KD
43KD
55KD
43KD
55KD
43KD

Figure S4

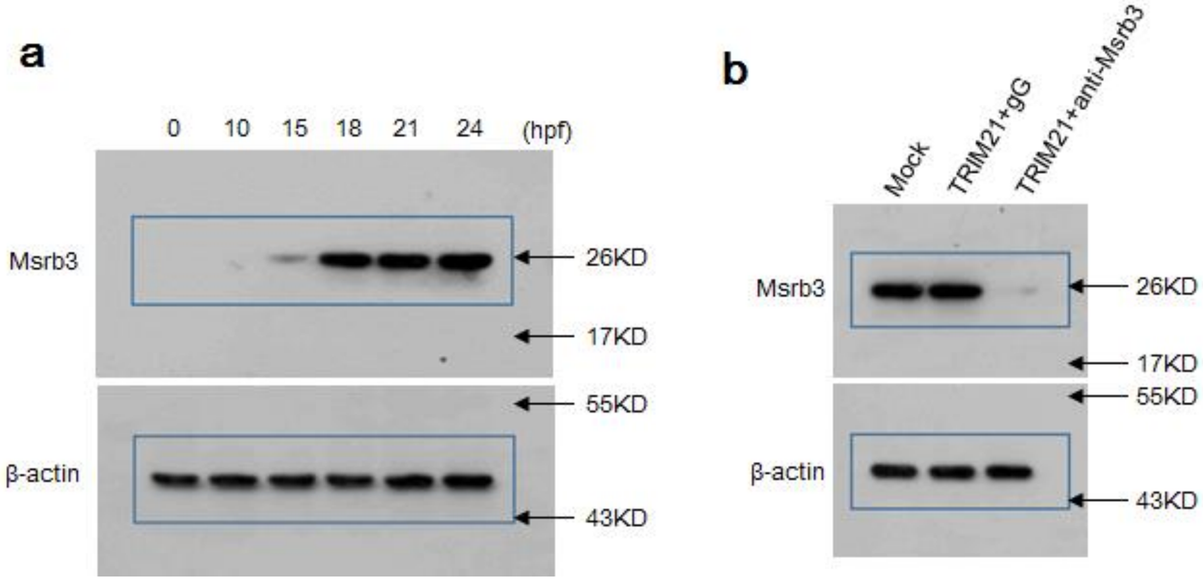

a
0
10
15
18
21
24
(hpf)
Msrb3
26KD
17KD
55KD
β-actin
43KD
b
Mock
TRIM21+gG
TRIM21+anti-Msrb3
Msrb3
26KD
17KD
55KD
β-actin
43KD

Figure S5

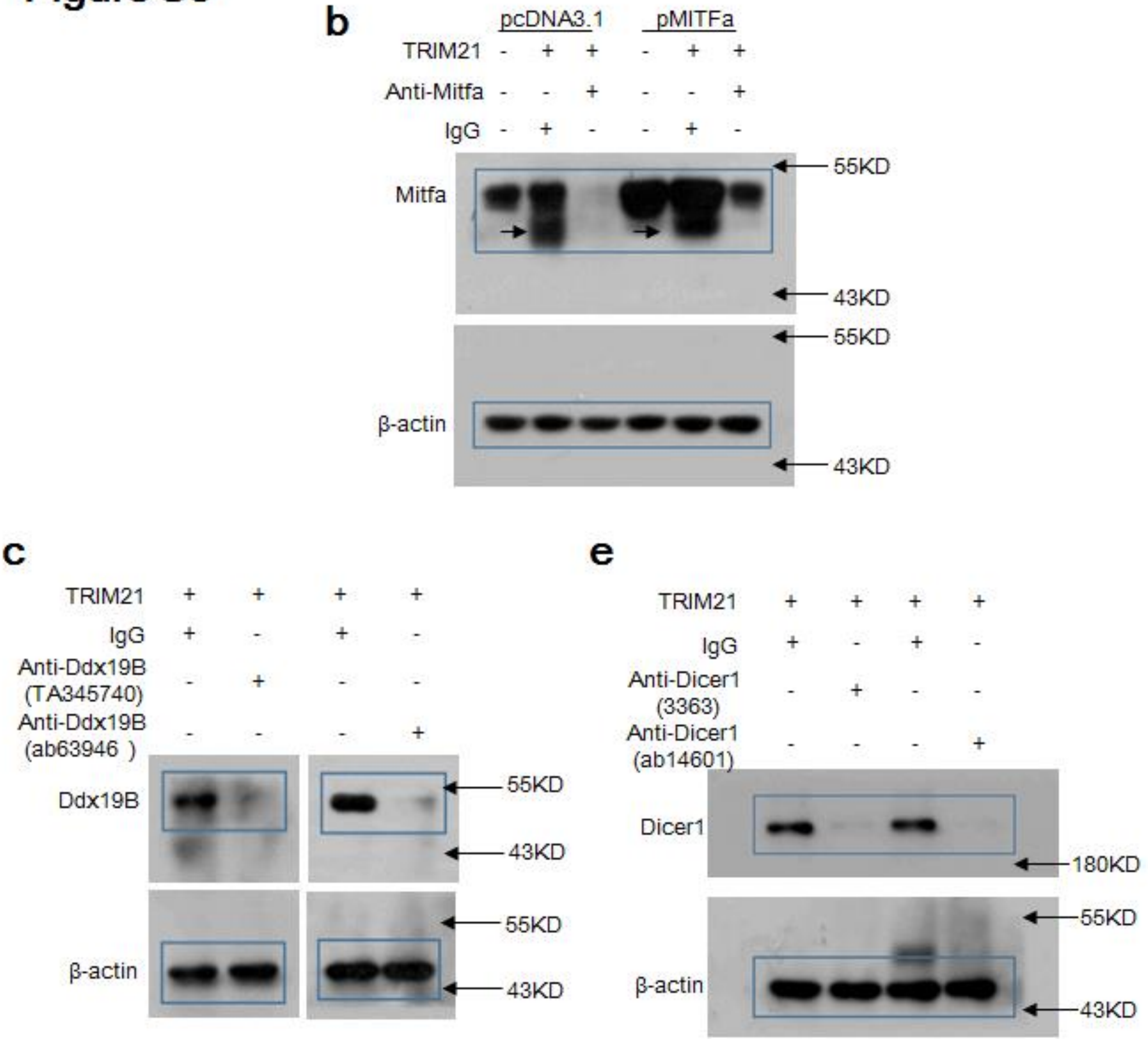

b
pcDNA3.1
pMITFa
TRIM21 - + + - + +
Anti-Mitfa - - + - - +
IgG - + - - + -
Mitfa
β-actin
55KD
43KD
c
TRIM21 + + + +
IgG + - + -
Anti-Ddx19B (TA345740) - + - -
Anti-Ddx19B (ab63946) - - - +
Ddx19B
β-actin
55KD
43KD
e
TRIM21 + + + +
IgG + - + -
Anti-Dicer1 (3363) - + - -
Anti-Dicer1 (ab14601) - - - +
Dicer1
β-actin
180KD
55KD
43KD

Figure S6

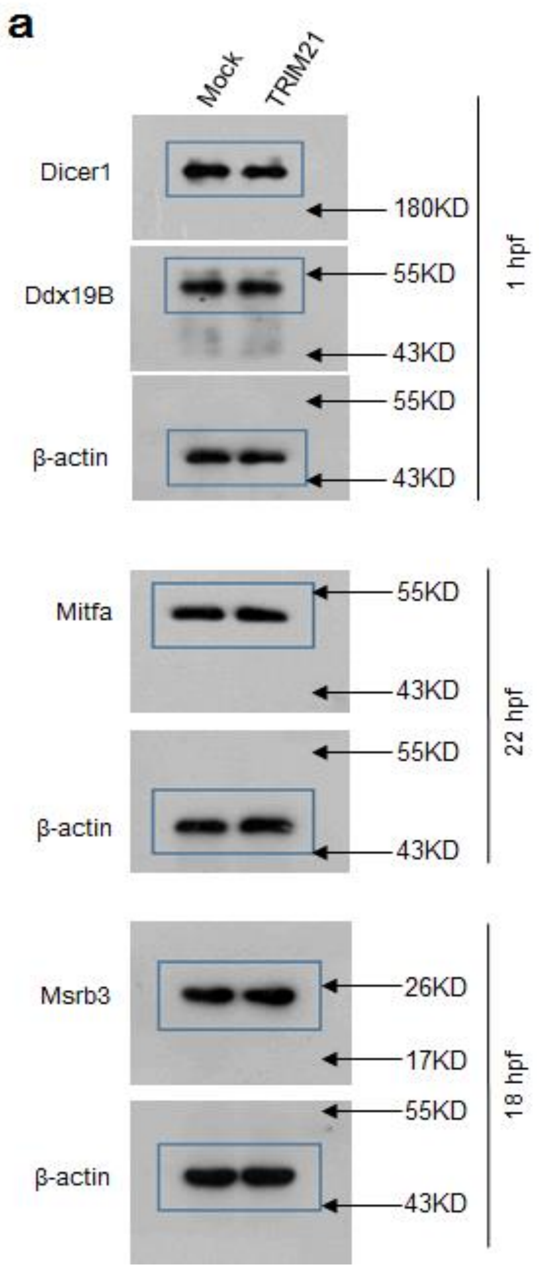
a
Mock
TRIM21
Dicer1
180KD
Ddx19B
55KD
43KD
β-actin
55KD
43KD
1 hpf
Mitfa
55KD
43KD
β-actin
55KD
43KD
22 hpf
Msrb3
26KD
17KD
β-actin
55KD
43KD
18 hpf

Figure S7

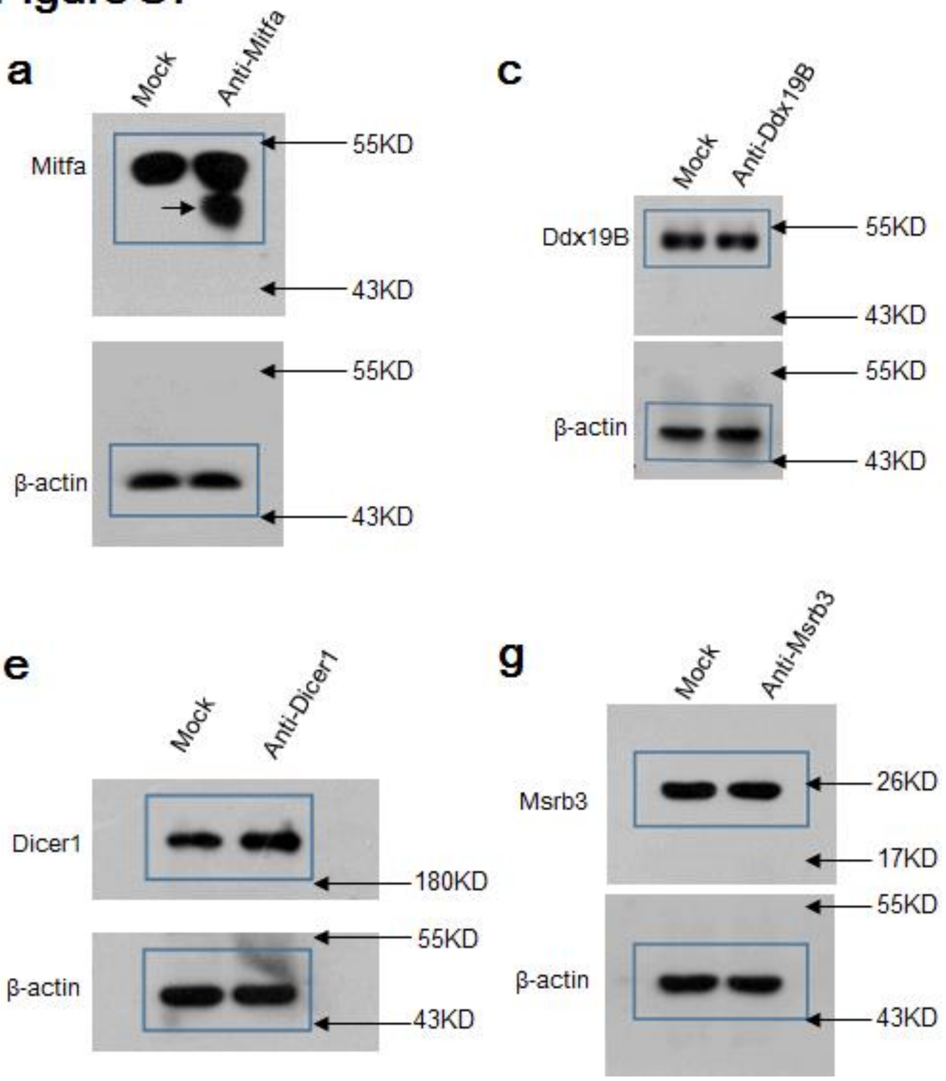
a
Mock
Anti-Mitfa
Mitfa
55KD
43KD
β-actin
55KD
43KD
c
Mock
Anti-Ddx19B
Ddx19B
55KD
43KD
β-actin
55KD
43KD
e
Mock
Anti-Dicer1
Dicer1
180KD
β-actin
55KD
43KD
g
Mock
Anti-Msrb3
Msrb3
26KD
17KD
β-actin
55KD
43KD

Figure S8

b

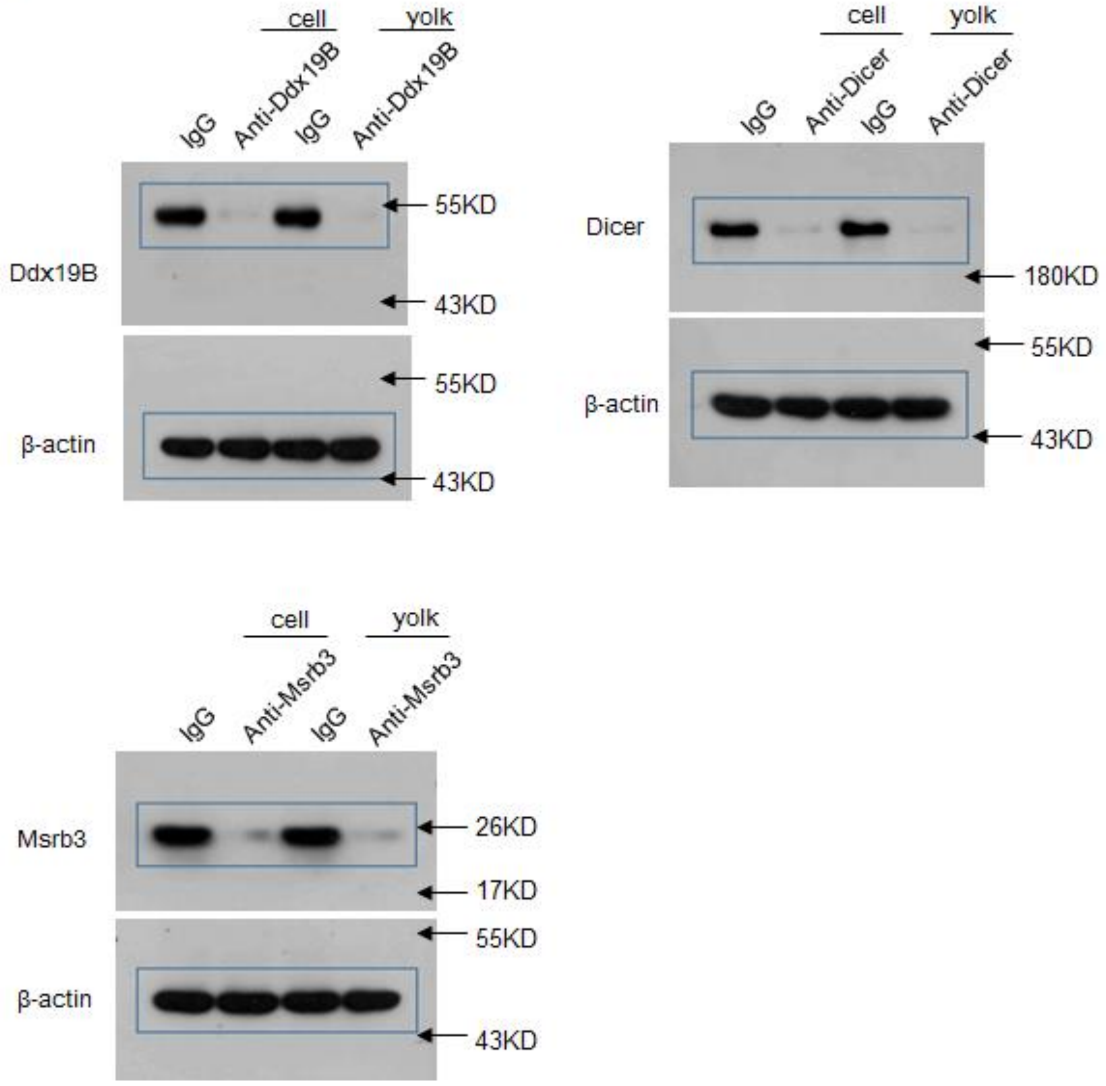
cell
yolk
IgG
Anti-Ddx19B
IgG
Anti-Ddx19B
Ddx19B
55KD
43KD
β-actin
55KD
43KD
cell
yolk
IgG
Anti-Dicer
IgG
Anti-Dicer
Dicer
180KD
β-actin
55KD
43KD
cell
yolk
IgG
Anti-Msrb3
IgG
Anti-Msrb3
Msrb3
26KD
17KD
β-actin
55KD
43KD
