# Supplementary material for: Degradation of endogenous proteins and generation of a null-like phenotype in zebrafish using Trim-Away technology
Source: Genome Biol. 2019 Jan 23;20:19. doi: 10.1186/s13059-019-1624-4 (PMC6343325; doi:10.1186/s13059-019-1624-4)
Supplement: Supplementary file 3 — Review history. (DOCX 744 kb) [file 13059_2019_1624_MOESM3_ESM.docx]

Review History

**First round of review**

**Reviewer 1**

**Comments to author:**

The manuscript by Chen et al reports a method for degradation of a target protein in vivo in zebrafish. The method presented in this manuscript uses E3 ubiquitin ligase, TRIM21, that binds to the Fc region of an antibody, while the variable region of the antibody binds to the target protein. As a result the protein complex gets degraded by the ubiquitin-proteasome pathway. 
This method could be useful in studying earlier embryonic phenotypes due to shorter duration of protein degradation. Authors, first injected antibody against EGFP, TRIM-21 in a transgenic line expressing EGFP, and showed the degradation of EGFP in a dose dependent manner as shown by the lack of fluorescence in injected embryos. One ng of TRIM21 and EGFP antibody degraded the EGFP protein the most. However, it is not clear whether increasing the dose of TRIM21 and antibody will have any effect on duration of protein degradation as it seems that both TRIM21 and antibody are depleted by 26 hpf. They further showed that proteins can be degraded several hours later than they expressed. Authors showed that targeting mitfa and ddx19b showed degradation of target proteins but the phenotype with mitfa downregulation was not comparable with mitfa morpholino at 28hpf, and the phenotypes gradually recovered at later stages showing that the technique may not be useful for studying phenotypes at later stages. Finally, authors presented data showing that maternally contributed proteins can also be degraded. 

Specific comments: 

Pg4, Line 20 – please define MG132 
Pg 5 Line 2: it is not clear whether authors generated stable transgenic line expressing TRIM-21 and EGFP or both components expressed transiently. 
Did authors notice any difference in degradation when antibodies are injected in cell vs yolk, for example, CRISPR/Cas9 shows dramatic effects when injected directly into one-cell stage embryos? 
A more detailed dosing experiment would be helpful to demonstrate the limit of the protein concentrations to be used. 
How much the human and zebrafish TRIM21 conserved? It would be interesting to test whether using zebrafish specific TRIM21 would have any effect on potency. 
Figure 1 B,C,D,E,F, G panels and supplementary figure S1 B,C,D,E,F, G looks same? 
Similarly other supplementary figures also has duplicated images from the main figures.

**Reviewer 2**

**Comments to author:**

Chen et al. report the use of Trim-Away to deplete proteins in zebrafish upon embryo injection with recombinant TRIM21 protein and IgG. The study demonstrates depletion of ectopically over expressed EGFP and endogenous proteins DDX19B, Dicer1 and Mitfa. Depletion occurs with similar kinetics as those previously reported in mammalian cells and with similar longevity. Depletion is nicely correlated with phenotypes in the zebrafish: For DDX19B, this resulted in smaller eyes in the first days post-fertilisation. For Dicer1, this caused morphogenesis defects and death within 4 days post-fertilisation. For Mitfa there were mild pigmentation defects. 

Specific Points: 

1. Particularly in Figure S1C but also in other blots in the supplementary figures, the images are cropped to an extent that information about protein size and blot specificity is lost. Given that this is supplementary data and space is not an issue, the entire blots should be presented either to replace the existing figure panels or as separate figures and with molecular weight markers clearly shown. 

2. Page 7, line 1: It would be helpful to plot the relative expression levels of TRIM21 and Mitfa vs time so the coincidence of degradation of the former upon expression of the latter is clear.

**Reviewer 3**

**Comments to author:**

In the submitted manuscript, Chen and colleagues port the Trim-Away approach to zebrafish. While obviously restricted to targeting proteins with existing functional antibodies, the approach using TRIM21 provides a new means to target proteins that are out of the grasp for traditional methods of morpholinos or zygotic mutants. 

The authors provide evidence for targeted degradation of several targeted proteins, most-impressively of mitfa. A key caveat of the presented work is the absence of any verification of potential systemic or otherwise unspecific effects on translation, protein stability, or overall gene expression - in particular the pleiotropic phenotypes of Ddx19b and Dicer targeting are difficult to distinguish from unspecific or toxic effects due to their severe impact on overall development. The targeting of such ubiquitously acting factors is a common issue with new methods in the field, as simply comparing morphometric data of treated vs control can be misleading without any molecular evidence (beyond successful targeting of the protein in question). 

The Mitfa targeting is much more clean and suggests faithful degradation of a targeted protein. Do the authors have any data on long-term effects in the TRIM21/anti-Mitfa injected embryos that could help readers weigh any long-term effects of the method? 

The authors should discuss the possibility of pleiotropic effects of the Trim-Away approach when used as injection-based method in zebrafish, in particular when demonstrated on broadly acting protein factors. Further, while experimentally out of the scope of the current manuscript, the authors should point out that transcriptome analysis of TRIM21-injected and control zebrafish (with and without antibody) would be instructive to identify such pleiotropic effects.\\springernature.com\london\Shares\BMC\GB\GENOME\CONTENT\PRIMARY\Tang (Trim-Away)\revised submission\Review History.docx

**Authors Response**

**Point-by-point responses to the reviewers’ comments:**

Reviewer #1 

The manuscript by Chen et al reports a method for degradation of a target protein in vivo in zebrafish. The method presented in this manuscript uses E3 ubiquitin ligase, TRIM21, that binds to the Fc region of an antibody, while the variable region of the antibody binds to the target protein. As a result the protein complex gets degraded by the ubiquitin-proteasome pathway. 
This method could be useful in studying earlier embryonic phenotypes due to shorter duration of protein degradation. Authors, first injected antibody against EGFP, TRIM-21 in a transgenic line expressing EGFP, and showed the degradation of EGFP in a dose dependent manner as shown by the lack of fluorescence in injected embryos. One ng of TRIM21 and EGFP antibody degraded the EGFP protein the most. However, it is not clear whether increasing the dose of TRIM21 and antibody will have any effect on duration of protein degradation as it seems that both TRIM21 and antibody are depleted by 26 hpf. They further showed that proteins can be degraded several hours later than they expressed. Authors showed that targeting mitfa and ddx19b showed degradation of target proteins but the phenotype with mitfa downregulation was not comparable with mitfa morpholino at 28hpf, and the phenotypes gradually recovered at later stages showing that the technique may not be useful for studying phenotypes at later stages. Finally, authors presented data showing that maternally contributed proteins can also be degraded. 

Specific comments: 

Pg4, Line 20 – please define MG132 

*[Response: MG132 is a well-known synthetic proteasome inhibitor widely used in research; therefore, we think that it is sufficient to define it as a proteasome inhibitor without going into details of its chemical nature.]* 

Pg 5 Line 2: it is not clear whether authors generated stable transgenic line expressing TRIM-21 and EGFP or both components expressed transiently. 

*[Response: We apologize for the confusion regarding this point. We used transgenic zebrafish transiently expressing EGFP; this information is provided in the revised manuscript (page 3, lines 13- 15).]* 

Did authors notice any difference in degradation when antibodies are injected in cell vs yolk, for example, CRISPR/Cas9 shows dramatic effects when injected directly into one-cell stage embryos? 

*[Response: We thank the Reviewer for the valuable comment. The injection of TRIM21/antibodies either into cells or yolks caused degradation of target proteins and induced similar phenotypic defects as indicated by the data presented in Fig. S8 of the revised manuscript. However, it is much easier to perform injection into yolks than into cells; therefore, we recommend the former (page 7, lines 20-22).]* 

A more detailed dosing experiment would be helpful to demonstrate the limit of the protein concentrations to be used. 

*[Response: We appreciate the Reviewer’s suggestion. Accordingly, we performed additional dosing experiments, which indicated that Trim-Away induced dose-dependent degradation of EGFP protein and reduction of the eye size in the range of 0.5–2 ng of TRIM21 and the target antibody (mixed 1:1) per embryo. However, the difference between effects caused by 1 and 2 ng was minimal, and the dosage increase above 2 ng did not cause further reduction of EGFP or eye size. Therefore, we recommend 1 ng TRIM21 and 1 ng antibody per embryo. We have discussed this issue in the revised manuscript (page 8, lines 1–6).]* 

How much the human and zebrafish TRIM21 conserved? It would be interesting to test whether using zebrafish specific TRIM21 would have any effect on potency. 

*[Response: Human and zebrafish TRIM21 proteins have 30% identity; their alignment is shown below. Our preliminary results indicate that co-injection of the zebrafish TRIM21 expression plasmid with an anti-EGFP antibody can repress EGFP expression. In the future, we plan to systematically compare the efficiency of the Trim-Away assay using TRIM21 proteins from different species. However, these data are out of the scope of the current article, which is a Short report and has strict space limitation. Therefore, we include these data below as “Data for reviewers only" rather than in the manuscript.]*

**Data for reviewers only**

**Acute degradation of EGFP triggered by zebrafish TRIM21 and anti-EGFP antibody in zebrafish embryos.**

**a** Alignment of human and zebrafish TRIM21 proteins. **b** The pEGFP-N3 plasmid and flag-tagged zebrafish *TRIM21* expression plasmid were injected into one-cell embryos to establish zebrafish transiently expressing EGFP and flag-tagged zebrafish TRIM21, the expression of flag-tagged zebrafish TRIM21 was determined by western blotting at 24 hpf. **c** Embryos co-expressing EGFP and flag-tagged zebrafish TRIM21 were injected with an anti-EGFP antibody or nonspecific IgG at 24 hpf, the expression of EGFP was determined by fluorescence microscopy at 24 and 24.5 hpf.

Figure 1 B,C,D,E,F, G panels and supplementary figure S1 B,C,D,E,F, G looks same? 
Similarly other supplementary figures also has duplicated images from the main figures. 

*[Response: Figure 1 has 6 panels (a–f) and shows the results of targeting endogenous Ddx19B and Dicer proteins, whereas supplementary Figure S1 has 7 panels (a–g) and shows the results of targeting EGFP. Thus, Figure 1 and supplementary Figure S1 are two different figures, which do not contain any duplicated information.]* 

Reviewer #2 

Chen et al. report the use of Trim-Away to deplete proteins in zebrafish upon embryo injection with recombinant TRIM21 protein and IgG. The study demonstrates depletion of ectopically over expressed EGFP and endogenous proteins DDX19B, Dicer1 and Mitfa. Depletion occurs with similar kinetics as those previously reported in mammalian cells and with similar longevity. Depletion is nicely correlated with phenotypes in the zebrafish: For DDX19B, this resulted in smaller eyes in the first days post-fertilisation. For Dicer1, this caused morphogenesis defects and death within 4 days post-fertilisation. For Mitfa there were mild pigmentation defects. 

Specific Points: 

1. Particularly in Figure S1C but also in other blots in the supplementary figures, the images are cropped to an extent that information about protein size and blot specificity is lost. Given that this is supplementary data and space is not an issue, the entire blots should be presented either to replace the existing figure panels or as separate figures and with molecular weight markers clearly shown. 

*[Response: We appreciate the Reviewer’s suggestion. In the revised manuscript, the entire blots are presented as separate supplementary figures, and molecular weight markers are clearly indicated. To improve the specificity of western blotting, we used two different anti-beta-actin antibodies to confirm equal protein loading: mouse and rabbit anti-beta-actin antibodies were used when the injected antibody was of the rabbit or mouse origin, respectively.]* 

2. Page 7, line 1: It would be helpful to plot the relative expression levels of TRIM21 and Mitfa vs time so the coincidence of degradation of the former upon expression of the latter is clear. 

*[Response: We thank the Reviewer for the valuable suggestion. In Figure 2c of the revised manuscript, we plotted the relative expression levels of TRIM21, IgG heavy chain, and Mitfa versus time. The goat anti-rabbit secondary IgG can detect not only the anti-Mitfa antibody bound to the Mitfa protein, but also the injected rabbit anti-Mitfa antibody. Since the molecular weight of the Mitfa protein is similar to that of the Ig heavy chain, the band corresponding to Mitfa is very close to that of the Ig heavy chain, and the efficiency of Mitfa degradation was obvious when the injected anti-Mitfa antibody completely disappeared.]* 

Reviewer #3 

In the submitted manuscript, Chen and colleagues port the Trim-Away approach to zebrafish. While obviously restricted to targeting proteins with existing functional antibodies, the approach using TRIM21 provides a new means to target proteins that are out of the grasp for traditional methods of morpholinos or zygotic mutants. 

The authors provide evidence for targeted degradation of several targeted proteins, most-impressively of mitfa. A key caveat of the presented work is the absence of any verification of potential systemic or otherwise unspecific effects on translation, protein stability, or overall gene expression - in particular the pleiotropic phenotypes of Ddx19b and Dicer targeting are difficult to distinguish from unspecific or toxic effects due to their severe impact on overall development. The targeting of such ubiquitously acting factors is a common issue with new methods in the field, as simply comparing morphometric data of treated vs control can be misleading without any molecular evidence (beyond successful targeting of the protein in question). 

The Mitfa targeting is much more clean and suggests faithful degradation of a targeted protein. Do the authors have any data on long-term effects in the TRIM21/anti-Mitfa injected embryos that could help readers weigh any long-term effects of the method? 

The authors should discuss the possibility of pleiotropic effects of the Trim-Away approach when used as injection-based method in zebrafish, in particular when demonstrated on broadly acting protein factors. Further, while experimentally out of the scope of the current manuscript, the authors should point out that transcriptome analysis of TRIM21-injected and control zebrafish (with and without antibody) would be instructive to identify such pleiotropic effects. 

*[Response: We appreciate these valuable suggestions. In the original manuscript, we provided the data confirming the specificity of the Trim-Away technology, which showed that restoration of Mitfa expression after co-injection of the Mitfa expression plasmid with TRIM21/anti-Mitfa antibody completely rescued the pigmentation phenotype, and that targeting the same protein by two different antibodies produced similar phenotypic defects. However, these data did not demonstrate the absence of nonspecific effects, especially for pleiotropic phenotypes of Ddx19b and Dicer. In the revised manuscript, we provide additional phenotypic analysis of a protein which is not ubiquitously required and does not exert pleiotropic effects. Methionine sulfoxide reductase B3 (Msrb3) is essential for the formation of otoliths, and we showed that Msrb3 knockdown in zebrafish embryos using Trim-Away resulted in the production of tiny, fused otoliths at abnormal numbers. These data are presented in supplementary Figure S4 in the revised manuscript (page 6, lines 20–22; page 7, lines 1-2). In addition, as suggested by the Reviewer, we pointed out that “To clarify pleiotropic or unspecific effects, transcriptome analysis should be performed to compare gene expression in Trim-Away embryos with that in zygotic or maternal-zygotic mutants.” (page 7, lines 16-19). 
Our data revealed that the injection of TRIM21 with an anti-Mitfa antibody into one-cell embryos may have no obvious long-term effects and that the injected embryos were able to develop into adult zebrafish, which were fertile and displayed no gross abnormalities. We have added this information in the revised manuscript (page 6, lines 16–18).]*

**Second round of review**

**Reviewer 3**

The authors have added significant new data to support the claims of their initial manuscript. A few minor changes would finalize the manuscript: 

1) the passage where mitfa and msrb3 seems to appear without proper tie-in with the previous data. One or two introductory sentences as to why these genes were chosen, etc. would greatly improve the manuscript's flow. 

2) the rescue of mitfa targeting using overexpression of mitfa is peculiar - this suggests that MITFA overexpression likely overwhelms the antibodies provided. The authors are encouraged to discuss this in the manuscript, as it provides an important consideration in the application of the TRIM21 technique.

**Authors Response**

**Point-by-point responses to the reviewers’ comments:**

Point-by-point responses to the comments of Reviewer #3 

The authors have added significant new data to support the claims of their initial manuscript. A few minor changes would finalize the manuscript: 

1) the passage where mitfa and msrb3 seems to appear without proper tie-in with the previous data. One or two introductory sentences as to why these genes were chosen, etc. would greatly improve the manuscript's flow. 

*[Response: We thank the Reviewer for the valuable comment and have revised the manuscript accordingly.]* 

2) the rescue of mitfa targeting using overexpression of mitfa is peculiar - this suggests that MITFA overexpression likely overwhelms the antibodies provided. The authors are encouraged to discuss this in the manuscript, as it provides an important consideration in the application of the TRIM21 technique. 

*[Response: We appreciate this valuable suggestion. Indeed, the rescue experiment indicates that Mitfa overexpression was too strong and overwhelmed the effect of antibodies and TRIM21, suggesting that more TRIM21 and antibodies should be provided when the targeted proteins are highly abundant. We have dissuced this issue in the revised manuscript.]*
